# Resolution *via* diastereomeric amides of enantiopure 1,4‐benzoxathian‐2‐ and 3‐carboxylic acids and determination of their configuration

**DOI:** 10.1002/chir.23474

**Published:** 2022-05-20

**Authors:** Valentina Straniero, Giulia Lodigiani, Lorenzo Suigo, Ermanno Valoti

**Affiliations:** ^1^ Department of Pharmaceutical Sciences Università degli Studi di Milano Milan Italy

**Keywords:** (*S*)‐phenylethylamine, 1,4‐benzoxathiane‐2‐carboxamide, 1,4‐benzoxathiane‐3‐carboxamide, ^1^H NMR comparison, absolute configuration determination, chiral HPLC, diastereomeric amides

## Abstract

1,4‐Benzoxathiane, 2‐ or 3‐substituted, is an important scaffold, and despite its presence in several therapeutic agents, it is chemically unexploited. Furthermore, only a few examples in literature report this moiety in its enantiopure form. Here, taking advantage to the formation of diastereomeric amides by using (*S*)‐phenylethylamine, which show significant differences in terms of ^1^H‐nuclear magnetic resonance (NMR) spectra and other physical chemical properties, we defined for the first time the absolute configuration of each amide, both 2‐ or 3‐substituted. Moreover, the diastereomeric amides were further hydrolyzed in acid conditions, letting to the achievement of the corresponding 1,4‐benzoxathian carboxylic acids.

## Graphical abstract

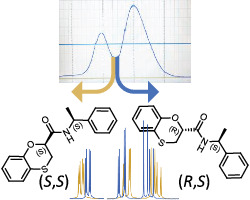

## 1 INTRODUCTION

**FIGURE 1:**
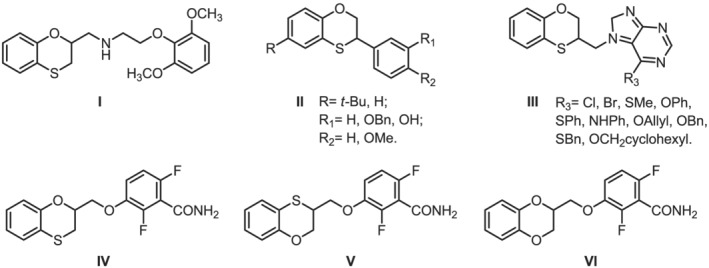
Literature 1,4‐benzoxathiane therapeutic agents, as antihypertensive compounds (**I**), sweeteners (**II**), anticancer agents (**III**), and antimicrobials (**IV** and **V**), deriving from 1,4‐benzodioxane FtsZ inhibitor **VI**

1,4‐Benzoxathiane is a key synthon of several therapeutic agents showing different applications. The first studied drugs including this moiety were the antihypertensive compounds^1^ and the sweeteners,^2^ whereas recently, anticancer agents^3^, ^4^, ^5^ and antimicrobials^6^ having this scaffold emerged (Figure 1). Specifically, while working on the development of antibacterial agents as FtsZ inhibitors, we prepared 2‐ and 3‐substituted 2,3‐dihydro‐1,4‐benzoxathianes linked to a 2,6‐difluorobenzamide moiety by a methylenoxy linker (compounds **IV** and **V** in Figure 1).

The development of compounds **IV** and **V** started from our considerations on how the 1,4‐benzodioxane system is well accepted by the FtsZ ligand binding site^7^, ^8^, ^9^, ^10^, ^11^ and from our latest computational model, which suggested us the inclusion of a sulfur atom as bioisoster of the oxygen one. As a result, we decided to move from compound **VI** (Figure 1), which was one of the most promising bactericidal antimicrobials, and to evaluate compounds **IV** and **V** in Figure 1.

We considered the established synthetic method used for the obtainment of 1,4‐benzodioxane derivatives and developed a completely innovative and easy method for the preparation of both 2‐ and 3‐substituted 2,3‐dihydro‐1,4‐benzoxathiine.^12^ We indeed demonstrated how the regioselectivity of this scaffold is strongly dependent on the polarity of the reaction medium as well as on the nature of the reagents. We decided to treat the 2‐mercaptophenol with triethylamine in a polar solvent mixture (acetonitrile/water: 1/1), in the presence of ethyl 2,3‐dibromopropionate. In these conditions, we obtained the ethyl 1,4‐benzoxathian‐2‐ and 3‐carboxylate with a 60% and 40% ratio, respectively (full experimental details and nuclear magnetic resonance (NMR) spectra are reported in the Supporting Information of our previous papers^6^, ^12^). The esters underwent different reactions to yield the desired compounds, which were tested for their antimicrobial properties. Surprisingly, we noticed how maintaining the oxygen in position 1—while having a sulfur atom in position 4—resulted in a fivefold most potent compound (**IV**), showing high minimum inhibitory concentrations (MICs) on both methicillin‐sensitive *Staphylococcus aureus* and methicillin‐resistant *S. aureus*.^6^

These interesting results, together with our previous considerations on how the absolute configuration of the compounds^7^ could influence the anti‐staphylococcal activity and with our expertise in the resolution of different chiral compounds, by the formation of diastereomeric salts^13^, ^14^, ^15^, ^16^ or by the covalent binding with enantiopure derivatives,^17^ moved us aiming the obtainment of the enantiopure forms of the 3‐(1,4‐benzoxathiane‐2‐yl)‐2,6‐difluorobenzamide **IV**. From the best of our knowledge, the chemistry of this scaffold is quite scarce,^18^, ^19^, ^20^, ^21^, ^22^, ^23^ and only few examples of chiral 1,4‐benzoxathianes are reported in literature.^1^, ^4^, ^24^ Moreover, a unique resolution method for the formation of (+) and (−) 1,4‐benzoxathian‐2‐carboxylic acids^24^ is known, and it does not include the 3‐substituted derivatives, records improvable yields, and does not disclose the absolute configuration of the obtained enantiopure carboxylic acids.

**SCHEME 1:**
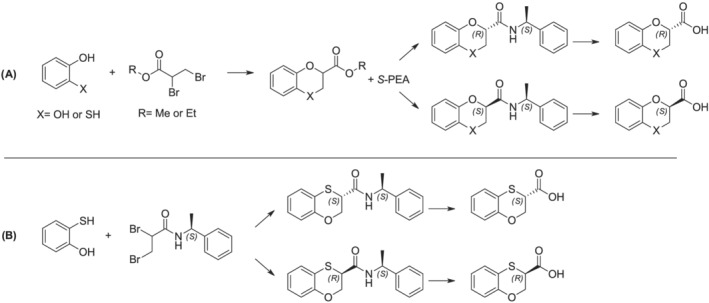
Synthetic overviews for the obtainment of enantiopure 1,4‐benzodioxan carboxylic acids and 1,4‐benzoxathian‐2‐carboxylic acids (A) or 1,4‐benzoxathian‐3‐carboxylic acids (B)

We thus considered the recent idea of Fumagalli and coworkers,^25^, ^26^ who took advantage by the covalent coupling of the resolving agent (the *S* enantiomer of the 1‐phenylethylamine: *S*‐PEA) with the two enantiomers (Scheme 1A).

We previously demonstrated how the treatment of methyl or ethyl 2,3‐dibromopropionate with 2‐mercaptophenol in presence of an organic base could let to the obtainment of the single 1,4‐benzoxathian‐2‐ carboxylate or to both the 1,4‐benzoxathian‐2‐ and 3‐carboxylate, following a solvent‐dependent trend.^12^ Moreover, in the same paper, we also observed that 2‐mercaptophenol treated in water with 2,3‐dibromopropionamide, which is indeed more polar than the corresponding esters seen before, could let the formation of the pure 1,4‐benzoxathian‐3‐carboxylamide.^12^

**FIGURE 2:**
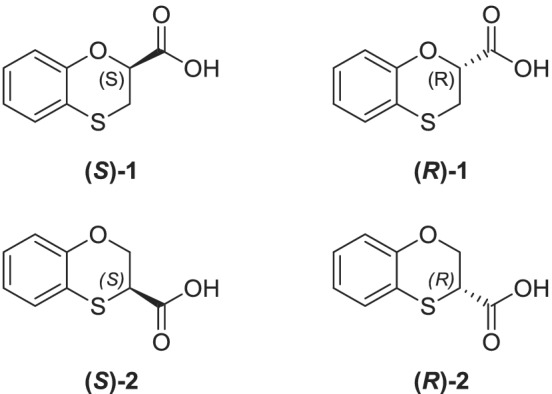
Enantiopure 1,4‐benzoxathian‐2‐(**(*S*)‐1** and **(*R*)‐1**) and 3‐(**(*S*)‐2** and **(*R*)‐2**) carboxylic acids, objects of the present paper

We thus decided to proceed separately for the formation of the pure diastereoisomeric amides of each regioisomer. On one side, by reacting the 2‐mercaptophenol with ethyl 2,3‐dibromopropionate in acetone, under reflux, we prepared the pure ethyl 1,4‐benzoxathian‐2‐carboxylate (Scheme 1A). The ester was quantitatively converted into the two diastereomeric *N*‐1‐phenylethylamides, by using *S*‐PEA, and the two amides further isolated by flash chromatography on silica gel and characterized. The enantiopure amides separately underwent acid hydrolysis, achieving the desired chiral acids **(*S*)‐1** and **(*R*)‐1** (Figure 2), showing high enantiomeric excesses.

The comparison and merging of chiral high‐performance liquid chromatography (HPLC) data of carboxylic acids and NMR spectra of amides let us define the absolute configurations of the final compounds **1**.

On the other side, considering what seen before with the 2,3‐dibromopropionamide,^12^ we tried directly treating the 2‐mercaptophenol with the 2,3‐dibromopropionamide of *S*‐PEA, ad hoc prepared, in a polar solvent (Scheme 1B). As a result, we obtained the two single 1,4‐benzoxathian‐3‐carboxylamides of *S*‐PEA, without detecting any traces of the 2‐regioisomer, neither in NMR spectra nor in HPLC analysis. This pair of amides was easily isolated on silica gel, too, and then characterized and their absolute configuration defined. Moreover, the amides further hydrolyzed in acid conditions, letting the achievement of the 1,4‐benzoxathian‐3‐carboxylic acid **2** (Figure 2).

With this work, we resulted in developing a reliable and easy method for the obtainment of a common and chemically unexploited scaffold. Moreover, for the first time in literature, we defined the configuration of each amide, by comparing 1,4‐benzoxathianes phenylethyl amides ^1^H NMR spectra, as well as other physical chemical properties, with benzodioxane ones.^26^ Moreover, we further confirmed the absolute configuration by transforming our carboxylic acid **(*R*)‐1** into the unique known chiral benzoxathiane scaffold: the 2‐hydroxymethyl‐1,4‐benzoxathiane.^4^, ^5^

## 2 MATERIALS AND METHODS

### 2.1 General

Reactants and reagents were purchased from different commercial suppliers (Fluorochem and Sigma Aldrich‐Merck) and used without further purification. The solvents were purchased from Sigma Aldrich‐Merck.

^1^H and ^13^C NMR spectra were taken on Varian 300 Mercury NMR spectrometer operating at 300 MHz for ^1^H NMR and 75 MHz for ^13^C NMR. Chemical shifts (*δ*) were reported in ppm relative to residual solvent as internal standard. Signal multiplicity was used according to the following abbreviations: s = singlet, d = doublet, dd = doublet of doublets, ddd = doublet of double doublets, t = triplet, dt = doublet of triplets, dq = doublet of quadruplets, qd = quadruplet of doublets, q = quadruplet, dt = doublet of quartets, m = multiplet, and bs = broad singlet.

Silica gel F_254_ was used in analytical thin‐layer chromatography (TLC) and silica gel (particle size 40–63 μm, Merck) in flash chromatography (utilizing Sepachrom Puriflash XS420 instrument); visualizations were accomplished with ultraviolet (UV) light (254 nm). Melting points were determined by DSC Q20 (TA INSTRUMENTS).

**SCHEME 2:**
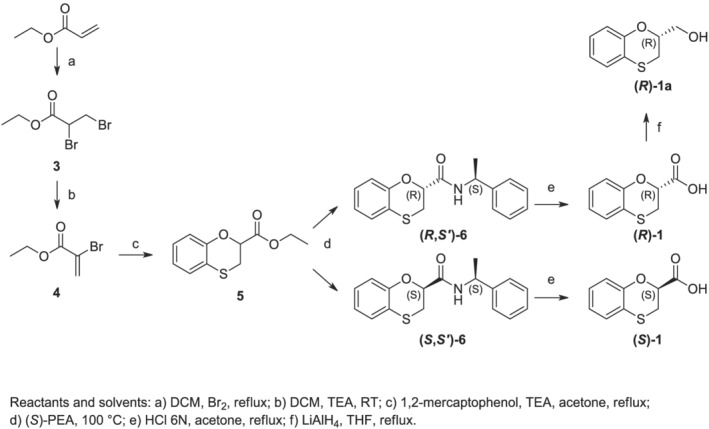
Detailed synthetic pathway for the preparation of enantiopure 2‐substituted 1,4‐benzoxathian derivatives

The HPLC analyses were performed by using Elite LaChrom HPLC system with diode array detector (190–400 nm). For the definition of regioisomer or diastereoisomer purity of **5** or of **6** and **10**, respectively (Scheme 1 and Scheme 2), we used a and a Water XBridgeTM C‐18 column (5 μm, 4.6 × 150 mm) and for the determination of the enantiomeric excess of final enantiomers of **1** and **2** a Phenomenex Lux® Cellulose‐1 column (3 μm, 4.6 × 150 mm). The four specific methods (A, B, C, and D) are reported in the Supporting Information and proved to be effective in separating the regioisomeric or diastereoisomeric pairs, as well as the enantiomers of the final compounds. High resolution mass spectrometry (HRMS) spectra were acquired on Q‐ToF SYNAPT G2‐Si HDMS 8K (Waters) coupled with an electrospray ionization (ESI) source in positive (ES+) or negative (ES−) ion mode.

### 2.2 HPLC analyses

Water XBridgeTM C‐18 column was flushed with the freshly prepared solution (acetate buffer (pH 4.7)/ACN (70:30, v/v) or ACN/water (70:30, v/v)) until column pressure was stable (Method A or B in the Supporting Information). Similarly, Phenomenex Lux® Cellulose‐1 column was flushed with *n*‐hexane/IPA (85:15, v/v) + 1.5% formic acid or *n*‐hexane/IPA (90:10, v/v) + 1.5% formic acid, when evaluating **1** or **2**, respectively (Methods C and D in the Supporting Information).

All the investigated samples were prepared through dissolution either of the crude reaction mixtures or of the purified products in the selected mobile phase, at the approximate concentrations of 1 mg/mL, filtered through a 0.45 μm filter and analyzed. The injection volume was 20 μL. Owing to the presence of the 1,4‐benzoxathiane scaffold in each compound, regioisomer content and enantiomeric excesses were evaluated on chromatograms recorded at 290 nm.

### 2.3 Synthesis

The preparation of the enantiopure 1,4‐benzoxathian‐2‐carboxylic acids was achieved as reported in the Scheme 2 below. We started following the preparation of the ethyl 2,3‐dibromopropionate **3** as done before,^12^ by brominating the commercially available ethyl acrylate in acetone under reflux. The consequent treatment in the same solvent with a single equivalent of TEA let the instant dehydrobromination and thus the achievement of the ethyl 2‐bromoacrylate **4**. The reaction of **4** with 2‐mercaptophenol and a further equivalent of TEA at room temperature in acetone under reflux let to accomplish the single regioisomer **5**.

The chromatographic purity of the ester **5** was evaluated by NMR and HPLC. Ethyl 1,4‐benzoxathian‐2‐carboxylate **(5)** was successfully completely converted into both amides **(*S*,*S′*)‐6** and **(*R*,*S′*)‐6** by dissolving it in 5 volumes of *S*‐PEA and refluxing the resulting reaction mixture. **(*S*,*S′*)‐6** and **(*R*,*S′*)‐6** were then easily separated on silica gel, and the diastereomeric amides showed also characteristic ^1^H NMR spectra, whose chemical shifts were totally diagnostic of the purity and the identity. Working parallelly on the two amides, **(*S*,*S′*)‐6** and **(*R*,*S′*)‐6** were converted into the desired enantiopure carboxylic acids **(*S*)‐1** and **(*R*)‐1** by acid hydrolysis.

Even if we know that the treatment of 2‐mercaptophenol with ethyl 2,3‐dibromopropionate and triethylamine in a polar solvent could let to the obtainment of both the ethyl 1,4‐benzoxathian‐2‐ and 3‐carboxylates, unfortunately, the application of the same reaction conditions for the preparation of the amide **10** brought to the complete degradation of the benzoxathiane 3‐substituted scaffold. This suggested us a liability of this moiety in basic conditions and moved us in evaluating an alternative synthesis.

**SCHEME 3:**
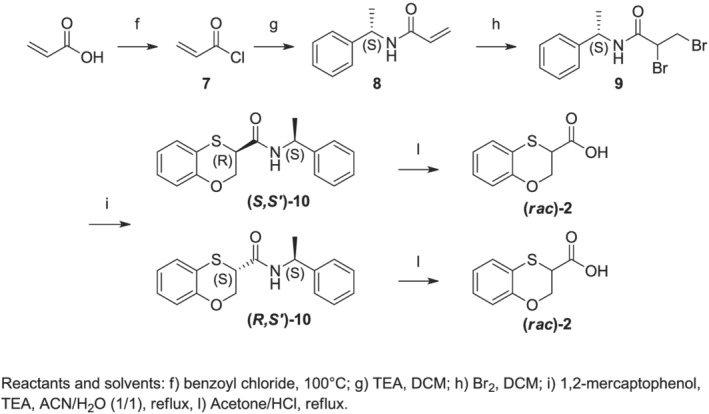
Detailed synthetic pathway for the preparation of enantiopure 3‐substituted 1,4‐benzoxathian derivatives

As a result, for the 1,4‐benzoxathian‐3‐carboxylic acids, we developed a different ad hoc synthetic pathway, which is displayed in Scheme 3. Commercial acrylic acid was quantitatively converted into the corresponding acyl chloride **7**, which was easily distilled from the reaction mixture, prior to the use with *S*‐PEA in DCM, thus achieving acrylamide **8**. The bromination of the double bond with bromine let us achieve intermediate **9**. The final reaction of *S*‐(PEA) dibromo propionamide **9** with 2‐mercaptophenol in an ACN/water mixture (20:80, v/v) let the obtainment, after chromatographic separation, of the two pure diastereomeric amides **(*S*,*S′*)‐10** and **(*R*,*S′*)‐10**. Also, these two amides showed distinct ^1^H NMR spectra, having chemical shifts able to quantitatively define their chiral purity. We aimed at the final formation of the two desired enantiopure carboxylic acids **(*S*)‐2** and **(*R*)‐2** by using the same acid hydrolysis seen for **(*S*)‐1** and **(*R*)‐1**. These conditions let us to achieve the desired carboxylic acids with good yields but unfortunately with the complete racemization of the compounds.

Since acids **1** and **2** showed different lipophilicity, we developed two different HPLC chiral methods for the enantiomeric excess quantification (see the Supporting Information for the complete details).

**Ethyl 2,3‐dibromopropionate** ^12^ **(3)**: 2.60 g (100%) of **3** as a yellowish oil was prepared, starting from ethyl acrylate (1.0 g, 10.03 mmol), as previously reported in our paper.^12^ ^1^H NMR (300 MHz, CDCl_3_): δ = 4.42 (dd, *J* = 11.4, 4.4 Hz, 1H), 4.29 (q, *J* = 7.1 Hz, 2H), 3.92 (dd, *J* = 11.4, 9.9 Hz, 1H), 3.67 (dd, *J* = 9.9, 4.4 Hz, 1H), 1.32 ppm (t, *J* = 7.1 Hz, 3H).

**Ethyl 2‐bromoacrylate** ^12^ **(4)**: 1.73 g (97%) of **4** as a yellow oil was prepared, starting from ethyl 2,3‐dibromopropionate (2.60 g, 10 mmol), as previously reported in our paper.^12^ ^1^H NMR (300 MHz, CDCl_3_): δ = 6.95 (s, 1H), 6.26 (s, 1H), 4.29 (q, *J* = 7.1 Hz, 2H), 1.34 ppm (t, *J* = 7.1 Hz, 3H).

**Ethyl 1,4‐benzoxathian‐2‐carboxylate (5)** ^12^: TEA (1.34 mL, 9.66 mmol) and **4** (1.73 g, 9.66 mmol) were dissolved in acetone (35 mL). 2‐Mercaptophenol (1.22 g, 9.66 mmol) was then slowly dropped. The reaction mixture was stirred at reflux for 18 h, then acetone was evaporated under pressure, and ethyl acetate and water were added to the aqueous phase. The organic phase was washed in sequence with 10% aqueous solution of NaCl, 10% aqueous solution of HCl, and 10% aqueous solution of NaHCO_3_. The organic phase was dried over Na_2_SO_4_, filtered and concentrated, to give a yellow oil as residue. The crude was purified by flash chromatography on silica gel, using Cyclohexane/Ethyl Acetate 95/5 as elution solvent, yielding **5** as a yellow oil. Yield: 1.62 g (75%). Retention time (Rt) (HPLC, Method A): 22.3 min. ^1^H NMR (300 MHz, CDCl_3_): δ = 7.04 (m, 2H), 6.96 (d, J = 7.7 Hz, 1H), 6.87 (t, J = 7.4 Hz, 1H), 4.99 (dd, *J* = 5.3, 3.8 Hz, 1H), 4.27 (q, *J* = 7.1 Hz, 2H), 3.29 (d, *J* = 3.8 Hz, 1H), 3.28 (d, *J* = 5.3 Hz, 1H), 1.28 ppm (t, J = 7.1 Hz, 3H). ^13^C NMR (75 MHz, CDCl_3_) δ: 168.7, 150.3, 127.5, 126.2, 121.8, 118.5, 116.5, 72.5, 61.9, 26.7, 14.1.

**(*2R*)‐*N*‐((*S*)‐1‐phenylethyl)‐2,3‐dihydrobenzo‐(1,4)‐oxathiine‐2‐carboxamide and (*2S*)‐*N*‐((*S*)‐1‐phenylethyl)‐2,3‐dihydrobenzo‐(1,4)‐oxathiine‐2‐carboxamide (6)**: A solution of ethyl 1,4‐benzoxathian‐2‐carboxylate (1.62 g, 7.24 mmol) in (*S*)‐1‐phenylethanamine (8.1 mL) was stirred at 100°C for 3 days, then it was diluted with ethyl acetate and washed with 10% aqueous solution of HCl and 10% aqueous solution of NaCl. The organic phase was dried over Na_2_SO_4_, filtered and concentrated, to give a yellow oil as residue. The crude was purified by flash chromatography on silica gel, using cyclohexane/ethyl acetate 95/5 as elution solvent, yielding **(*R,S′*)‐6** as the first eluted compound and **(*S,S′*)‐6** as the former one. Cumulative yield: 1.34 g (65.4%).

**(*R*,*S′*)‐6**: waxy‐oil; [α]_*D*_^25^ (c 1, CHCl_3_) = −61.2; Rt (HPLC, Method B): 7.20 min; ^1^H NMR (300 MHz, CDCl_3_) δ: 7.42–7.27 (m, 5H), 7.09 (m, 1H), 7.02 (m, 1H), 6.91 (m, 2H), 6.82 (d, *J* = 7.0 Hz 1H), 5.19 (dq, *J* = 7.0, 6.9 Hz 1H), 4.72 (dd, *J* = 8.4, 2.4 Hz, 1H), 3.42 (dd, *J* = 13.1, 2.4 Hz, 1H), 3.22 (dd, *J* = 13.1, 8.4 Hz, 1H), 1.51 (d, *J* = 6.9 Hz, 3H). ^13^C NMR (75 MHz, CDCl_3_) δ: 167.6, 149.9, 142.5, 128.8, 127.8, 127.6, 126.2, 126.0, 122.4, 118.3, 118.2, 74.5, 48.7, 27.5, 21.8. HRMS (TOF ES+, Na‐adduct): *m*/*z* 322.0879, 323.0907, 324.0864. Calculated mass 322.0878, evaluated mass 322.0879.

**(*S,S′*)‐6**: yellowish solid mp 108.9 °C; [α]_*D*_^25^ (c 1, CHCl_3_) = +95.7; Rt (HPLC, Method B): 6.71 min; ^1^H NMR (300 MHz, CDCl_3_) δ: 7.37–7.24 (m, 5H), 7.07 (m, 1H), 7.03 (m, 1H), 6.92 (d, *J* = 7.3 Hz, 1H), 5.19 (dq, *J* = 7.3, 6.8 Hz 1H), 4.77 (dd, *J* = 8.3, 2.3 Hz, 1H), 3.38 (dd, *J* = 13.2, 2.4 Hz, 1H), 3.17 (dd, *J* = 13.2, 8.3 Hz, 1H), 1.57 (d, *J* = 6.8 Hz, 3H). ^13^C NMR (75 MHz, CDCl_3_) δ: 167.7, 149.9, 142.6, 128.7, 127.8, 127.4, 126.0, 125.9, 122.4, 118.3, 118.2, 74.5, 48.5, 27.5, 21.9. HRMS (TOF ES+, Na‐adduct): *m*/*z* 322.0878, 323.0907, 324.0864. Calculated mass 322.0878, evaluated mass 322.0878.

**(2*R*)‐2,3‐dihydrobenzo‐(1,4)‐oxathiine‐2‐carboxylic acid (*R*‐1)**: The solution of **(*R,S*)‐6** (0.5 g, 1.76 mmol) in acetone (5 mL) was added of 50 mL of 6N aqueous solution of HCl, and the reaction kept refluxing overnight. The acetone was evaporated under reduced pressure, and the aqueous phase extracted with ethyl acetate. The organic phase was then washed with a 10% aqueous solution NaHCO_3_, so the aqueous phase was then acidified and extracted (3×) with ethyl acetate. The collected organic phases were dried over Na_2_SO_4_, filtered and concentrated, to give **(*R*‐1)** as white solid. Yield: 0.31 g (90%). mp 113.5°C, [α]_*D*_^25^ (c 1, CHCl_3_) = −100.3. Rt (HPLC, Method C): 15.43 min; ^1^H NMR (300 MHz, CDCl_3_) δ: 10.68 (bs, 1H), 7.17–6.99 (m, 2H), 6.99–6.83 (m, 2H), 5.07 (dd, *J* = 5.6, 3.4 Hz, 1H), 3.40–3.13 (m, 2H). ^13^C NMR (75 MHz, CDCl_3_) δ: 174.3, 149.9, 127.7, 126.4, 122.1, 118.4, 116.5, 72.2, 26.4. HRMS (TOF ES−): *m*/*z* 125.0059, 151.0219, 152.0249, 153.0176, 195.0116, 196.0146, 197.1541, 262.9990, 413.0133. Calculated mass 195.0116, evaluated mass 195.0116.

**(2*S*)‐2,3‐dihydrobenzo‐(1,4)‐oxathiine‐2‐carboxylic acid (*S*‐1)**: **(*S*‐1)** was obtained from **(*S*,*S*)‐6**, following the same procedure of **(*R*‐1)**, achieving **(*S*‐1)** as a white solid. Yield, mp, ^1^H and ^13^C are identical to the ones of **(*R*‐1)**, [α]_*D*_^25^ (c 1, CHCl_3_) = +100.2. Rt (HPLC, Method C): 19.7 min. HRMS (TOF ES−): *m*/*z* 123.9981, 125.0058, 151.0218, 152.0248, 153.0175, 195.0116, 196.0146, 197.1539, 255.0845, 413.0132. Calculated mass 195.0116, evaluated mass 195.0116.

**(2*R*)‐2,3‐dihydrobenzo‐(1,4)‐oxathiine‐2‐methanol** ^3^ **(*R*‐1a)**: Operating under a nitrogen atmosphere, a suspension of LiAlH_4_ (0.018 g, 0.5 mmol) in THF (5 mL) was added dropwise at 0°C of a solution of **(*R*‐1)** (0.05 g, 0.25 mmol) in THF. The reaction mixture was then warmed to RT and then refluxed for 3 h. After completion, the reaction mixture was quenched with ethyl acetate/water, then the organic phase was washed with 10% aqueous solution of HCl and 10% aqueous solution of NaCl. The organic phase was dried over Na_2_SO_4_, filtered and concentrated, to give a yellow oil as residue. Yield: 30 mg (66%). [α]_*D*_^25^ (c 1, CHCl_3_) = +33.7. ^1^H NMR (300 MHz, CDCl_3_) δ: 7.10–6.80 (m, 4H), 4.28 (m, 1H), 4.00–3.75 (m, 2H), 3.13 (dd, *J* = 13.2, 8.8 Hz, 1H), 2.95 (dd, *J* = 13.2, 2.2 Hz, 1H).

**Acryloyl chloride (7)**: A solution of acrylic acid (10.0 g, 137.62 mmol) in benzoyl chloride (40 mL) was left refluxing for 3 h. Afterwards the reaction mixture was distillated at 200°C, achieving pure **7** as a colorless oil. Yield: 9.90 g (80%). ^1^H NMR (300 MHz, CDCl_3_) δ: 6.64 (d, *J* = 16.6 Hz, 1H), 6.34 (dd, *J* = 16.6, 10.1 Hz, 1H), 6.17 (d, *J* = 10.1 Hz, 1H).

**(*S*)‐N‐(1‐phenylethyl)acrylamide (8)**: A solution of **7** (9.55 g, 105.51 mmol) in DCM was added dropwise at 0°C to a solution of triethylamine (17.58 mL, 126.61 mmol) and (*S*)‐1‐phenylethylamine (14.64 mL, 116.06 mmol) in DCM. The reaction mixture was left stirring overnight and then the organic phase was washed with 10% aqueous solution HCl, 10% aqueous solution NaHCO_3_ and 10% aqueous solution NaCl. The organic phase dried over Na_2_SO_4_, filtered and concentrated, to give **8** as yellowish solid. Yield: 14.64 g (72%). mp 174.7°C; [α]_*D*_^25^ (c 1, CHCl_3_) = −168.67. ^1^H NMR (300 MHz, CDCl_3_) δ: 7.38–7.23 (m, 5H), 6.29 (dt, *J* = 17.0, 1.4 Hz, 1H), 6.08 (ddd, *J* = 17.0, 10.2, 1.5 Hz, 1H), 5.83 (s, 1H), 5.64 (dt, *J* = 10.2, 1.5 Hz, 1H), 5.28–5.14 (m, 1H), 1.53 (dd, *J* = 6.9, 1.7 Hz, 3H).

**2,3‐dibromo‐N‐((*S*)‐1‐phenylethyl)propanamide (9)**: To a solution of **8** (5.15 g, 29.4 mmol) in DCM was added dropwise a solution of Bromine (1.5 mL, 29.4 mmol) to give a strong red solution. After 3 h, the solution faded into a pale yellow color and was quenched with a 10% aqueous solution Na_2_S_2_O_5_ until the phases faded completely. The phases were separated and the organic one dried over Na_2_SO_4_, filtered and concentrated, to give **9** as pale‐yellow solid. Yield: 8.87 g (90%). mp 200.6°C; [α]_*D*_^25^ (c 1, CHCl_3_) = −52.01. ^1^H NMR (300 MHz, CDCl_3_) δ: 7.41–7.20 (m, 1H), 6.37 (s, 1H), 5.14 (qd, *J* = 7.0, 1.4 Hz, 1H), 4.46 (ddd, *J* = 9.4, 4.5, 3.1 Hz, 1H), 3.97 (ddd, *J* = 10.3, 8.2, 3.9 Hz, 1H), 3.82 (ddd, *J* = 12.0, 10.3, 4.5 Hz, 1H), 1.54 (d, *J* = 7.0 Hz, 1H).

**(3*R*)‐N‐((*S*)‐1‐phenylethyl)‐2,3‐dihydrobenzo‐(1,4)‐oxathiine‐3‐carboxamide or (3*S*)‐N‐((*S*)‐1‐phenylethyl)‐2,3‐dihydrobenzo‐(1,4)‐oxathiine‐3‐carboxamide (10)**: TEA (4.57 mL, 32.83 mmol) and **9** (5 g, 14.92 mmol) were dissolved in 67 mL of a mixture of acetonitrile and water (20:80, v/v). 2‐Mercaptophenol (1.71 g, 13.56 mmol) was then slowly dropped. The reaction mixture was stirred at reflux for 72 h, then the solvents were evaporated under pressure, and ethyl acetate was added. The organic phase was washed in sequence with 10% aqueous solution of NaCl, 10% aqueous solution of HCl and 10% aqueous solution of NaHCO_3_. The organic phase was dried over Na_2_SO_4_, filtered and concentrated, to give a yellow oil as residue. The crude was purified by flash chromatography on silica gel, using cyclohexane/ethyl acetate 9/1 as elution solvent, yielding the former eluted compound that is supposed to be the **(*S,S′*)‐10** and the former one, which is supposed to be the **(*R,S′*)‐10**. Cumulative yield: 1.76 g (46.0%).

**Supposed (*S,S′*)‐10** white solid mp 116.3°C. [α]_*D*_^25^ (c 1, CHCl_3_) = −88.7; Rt (HPLC, Method B): 6.04 min;^1^H NMR (300 MHz, CDCl_3_) δ: 7.41–7.19 (m, 2H), 7.18–7.00 (m, 1H), 7.03–6.82 (m, 1H), 5.09 (dq, *J* = 7.0, 6.98 Hz, 1H), 4.96 (dd, *J* = 11.2, 3.4 Hz, 1H), 4.22 (dd, *J* = 11.2, 2.5 Hz, 1H), 3.89 (dd, *J* = 3.4, 2.5 Hz, 1H), 1.36 (d, *J* = 7.0 Hz, 1H). ^13^C NMR (75 MHz, CDCl_3_) δ: 167.0, 151.9, 142.6, 128.7, 127.4, 127.1, 126.3, 126.0, 122.2, 118.9, 115.1, 66.4, 49.5, 43.1, 21.7. HRMS (TOF ES+, Na‐adduct): *m*/*z* 322.0880, 323.0908, 324.0864. Calculated mass 322.0878, evaluated mass 322.0880.

**Supposed (*R,S′*)‐10** white solid mp 140.0°C. [α]_*D*_^25^ (c 1, CHCl_3_) = −9.7; Rt (HPLC, Method B): 5.72 min; ^1^H NMR (300 MHz, CDCl_3_) δ: 7.16 (dt, *J* = 6.5, 2.3 Hz, 1H), 7.07 (ddd, *J* = 7.6, 3.3, 1.3 Hz, 1H), 6.91–6.81 (m, 1H), 5.11 (dq, *J* = 7.3, 7.0 Hz, 1H), 4.97 (ddd, *J* = 11.2, 3.5, 1.1 Hz, 1H), 4.24 (ddd, *J* = 11.2, 2.2, 1.2 Hz, 1H), 3.93 (ddd, *J* = 3.4, 2.3, 1.1 Hz, 1H), 1.46 (d, *J* = 7.0 Hz, 1H). ^13^C NMR (75 MHz, CDCl_3_) δ: 166.9, 151.9, 142.5, 128.5, 127.3, 127.0, 126.5, 125.5, 122.2, 118.9, 66.6, 49.1, 43.1, 21.9. HRMS (TOF ES+, Na‐adduct): m/z 322.0882, 323.0909, 324.0866. Calculated mass 322.0878, evaluated mass 322.0882.

**2,3‐dihydrobenzo‐(1,4)‐oxathiine‐3‐carboxylic acid (*rac*‐2)**: The solution of **(*S,S*)‐10** or **(*R,S*)‐10** (0.5 g, 1.76 mmol) in acetone (5 mL) was added of 50 mL of 6N aqueous solution of HCl, and the reaction kept refluxing overnight. The acetone was evaporated under reduced pressure and the aqueous phase extracted with ethyl acetate. The organic phase was then washed with a 10% aqueous solution NaHCO_3_, the aqueous phase was then acidified and extracted (3×) with ethyl acetate. The collected organic phases were dried over Na_2_SO_4_, filtered and concentrated, to give **(*rac*‐2)** as white solid. Yield: 0.31 g (90%). mp 112.8°C. HPLC (Method D): Rt = 17.19 min (*R*) and 18.25 min (*S*). ^1^H NMR (300 MHz, CDCl_3_) δ: 10.15 (bs, 1H), 7.21–6.96 (m, 1H), 6.95–6.78 (m, 1H), 4.60 (dd, *J* = 11.5, 5.5 Hz, 1H), 4.48 (dd, *J* = 11.6, 2.7 Hz, 1H), 4.14 (dd, *J* = 5.5, 2.8 Hz, 1H). ^13^C NMR (75 MHz, CDCl_3_) δ: 174.7, 150.9, 126.9, 126.1, 122.2, 118.5, 116.8, 65.6, 41.2. HRMS (TOF ES−): *m*/*z* 123.9980, 125.0058, 151.0216, 152.0247, 153.0174, 195.0114, 197.1539, 198.1572, 413.0130. Calculated mass 195.0116, evaluated mass 195.0114.

## 3 RESULTS AND DISCUSSION

While designing the synthetic protocol described in the specific paragraph, we initially thought to follow a single pathway, by treating methyl or ethyl 2,3‐dibromopropionate with 2‐mercaptophenol in a polar solvent, to obtain the two regioisomers, and then by transforming the esters into the corresponding amides. Nevertheless, together with the quantitative conversion of the 2‐substituted ester into the corresponding amides, we observed the complete chemical instability of benzoxathiane 3‐substituted carboxylate in (*S*)‐PEA. This moved us in evaluating an alternative strategy, starting from what we recently noted while reacting in water the 2‐mercaptophenol with 2,3‐dibromopropionamide. In that case, we resulted in achieving the pure 1,4‐benzoxathian‐3‐carboxylamide.^12^

As a result, we treated the 2‐hydroxythiophenol with (*S*)‐2,3‐dibromo‐1‐phenylethyl propanamide in a mixture of ACN/water because the use of pure water did not let to the complete solubilization of the two reactants. Nevertheless, we fortunately resulted in obtaining the two diastereomeric amides, exclusively of the 3‐regioisomer. The chemical instability of the benzoxathiane 3‐substituted scaffold turned out to be useful in developing two completely regiocontrolled syntheses. We thus obtained the four diastereomeric amides, following the two peculiar pathways reported in Schemes 1 and 2, and we could easily purify them by flash chromatography on silica gel, thanks to their differences in polarity, and thus in chromatographic behavior. Once we have isolated the four amides, totally unexplored before, we completely characterize them, by using NMR, HPLC, differential scanning calorimetry (DSC) and optical rotation, aiming at the determination of the absolute configuration of each amide.

All the obtained characterization data are completely innovative in literature, so we decided to compare the benzoxathiane properties with the ones of the two diastereomeric *N*‐(1′‐phenylethyl)‐1,4‐benzodioxane‐2‐carboxamides, prepared by Fumagalli and coworkers from methyl 1,4‐benzodioxane carboxylate and (*R*)‐PEA.

**FIGURE 3:**
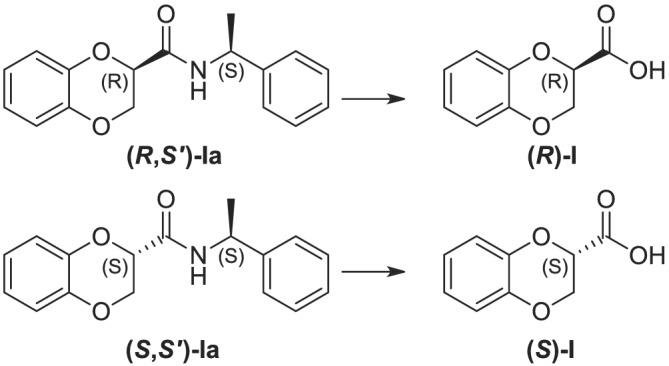
Known 1,4‐benzodioxane derivatives

We followed the procedure reported in their paper and synthetized the two benzodioxane carboxamides (Compounds **(*R*,*S*′)‐Ia** and **(*S*,*S*′)‐Ia** in Figure 3), using (*S*)‐PEA as for benzoxathiane derivatives. We then further complete their characterization, evaluating the chromatographic features (Tables 1 and 2), the ^13^C NMR,^27^, ^28^ and the optical rotations (Table 1), because these data were not determined before. Once we had all the data in hands, we compared the physical–chemical features of the six amides, which are reported in Tables 1 and 2, to hypothesize the absolute configuration of the novel benzoxathiane amides.

**TABLE 1:** Physical–chemical features of the six amides

Compound	Rf (TLC)	Rt (HPLC)	mp	Optical rotation
**(*S*,*S*′)‐Ia**	Higher	Lower	75.0°C^25^	−41.4
**(*R*,*S*′)‐Ia**	Lower	Higher	102.7°C^25^	+71.8
**(*R*,*S*′)‐6**	Higher	Lower	Wax	−61.2
**(*S*,*S*′)‐6**	Lower	Higher	108.9°C	+95.7
**(*S*,*S*′)‐10**	Higher	Lower	116.3°C	+88.7
**(*R*,*S*′)‐10**	Lower	Higher	140.0°C	−9.7

Abbreviations: HPLC, high‐performance liquid chromatography; Rf, retention factor; Rt, retention time; TLC, thin‐layer chromatography.

**TABLE 2:** ^1^H NMR data of the six amides

Compound	^1^H NMR chemical shifts					
	C(2)		C(3)		CH (PEA)	CH_3_ (PEA)
**(*S*,*S*′)‐Ia**	4.61		4.57 (cis)	4.19 (trans)	5.18	1.49
**(*R*,*S*′)‐Ia**	4.71		4.52 (cis)	4.15 (trans)	5.18	1.55
**(*R*,*S*′)‐6**	4.72		3.42 (cis)	3.22 (trans)	5.20	1.51
**(*S*,*S*′)‐6**	4.77		3.38 (cis)	3.17 (trans)	5.20	1.57
**(*S*,*S*′)‐10**	4.96 (trans)	4.22 (cis)	3.89		5.11	1.36
**(*R*,*S*′)‐10**	4.97 (trans)	4.24 (cis)	3.93		5.09	1.46

Abbreviation: NMR, nuclear magnetic resonance.

Firstly, we considered the chromatographic features e compared the TLC and HPLC data of each amide. The chromatographic purification on silica gel was easily achieved for each couple of diastereoisomers, because the retention factors (Rfs) were quite distant, while using common elution mixtures of cyclohexane/ethyl acetate, differently proportioned. After the isolation, the amides were characterized and HPLC evaluated. When comparing benzodioxane (**(*S*,*S*′)‐Ia** and **(*R*,*S*′)‐Ia**) with 2‐ and 3‐substituted benzoxathiane amide (**6** and **10**, respectively) chromatographic data, we soon noticed a similar trend: The amide having the higher TLC Rf was indeed the secondly eluted in HPLC, thus suggesting a comparable polarity, due to the structural analogy of the chiral center in alpha‐position of the carboxylic acid.

**FIGURE 4:**
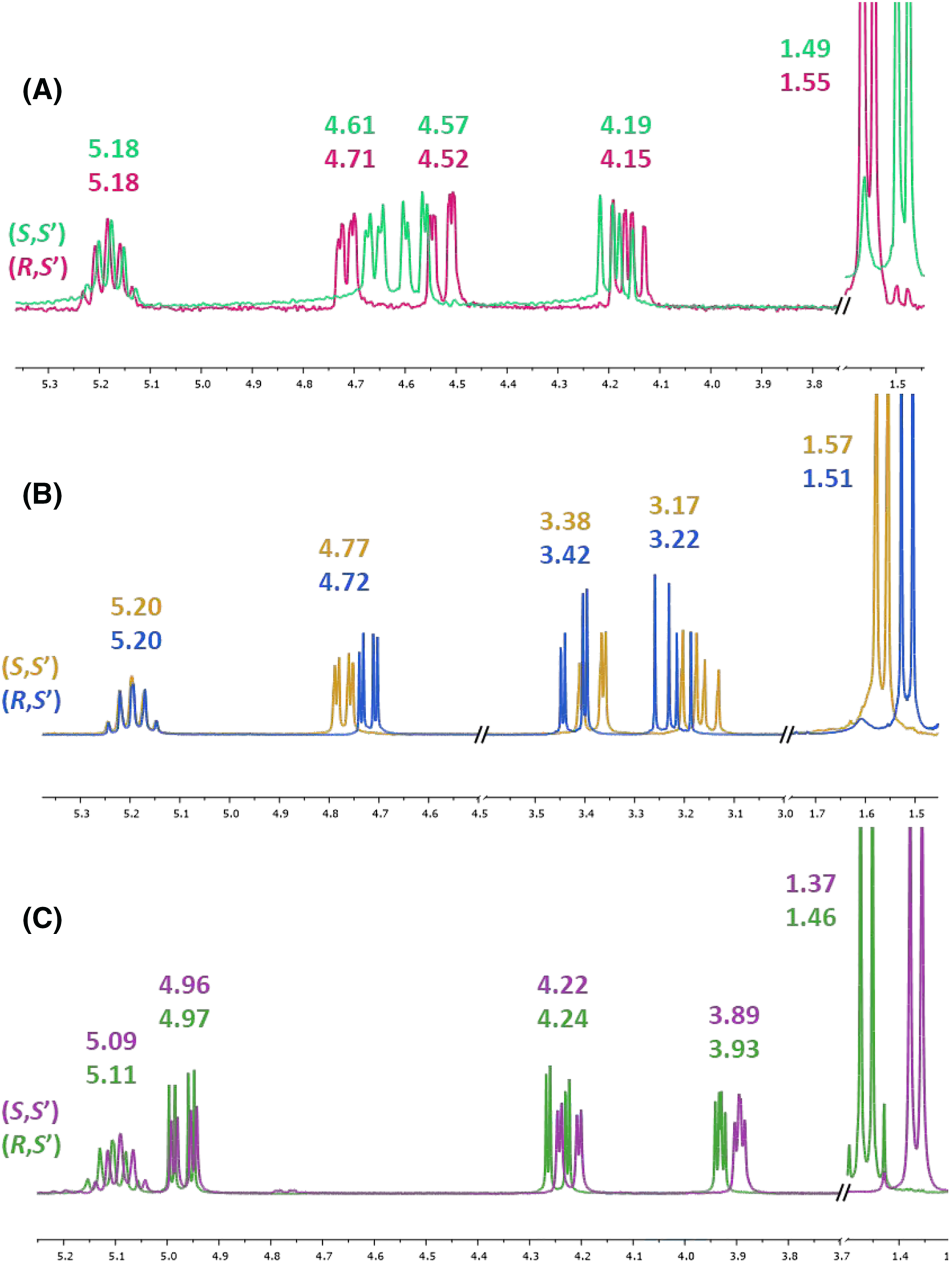
^1^H nuclear magnetic resonance (NMR) spectra comparison of 1,4‐benzodioxane carboxamides (A), 1,4‐benzoxathiane‐2‐carboxamides (B), and 1,4‐benzoxathiane‐3‐carboxamides (C)

We then moved in comparing the ^1^H NMR spectra of the three diastereomeric couples of amides (Table 2), searching for similarities or discrepancies in terms of chemical shifts and coupling constants of the proton/protons on C(2) and C(3) (Figure 4).

As reported in Table 2 and Figure 4, the chemical shift of the CH(2), the one on the benzodioxane chiral center, was strongly different between the two amides. (*S*,*S*′), the less polar amide among the two benzodioxane ones, shows a lower chemical shift (4.61), which differs from the one of (*R*,*S*′) of 0.10 ppm (4.71) (Figure 4A). An analogous trend was seen with 2‐ benzoxathiane amides (Figure 4B): The higher compound in TLC chromatography was also the one having the lower CH(2) chemical shift. Also in this case, the difference between the two amide values is quite high (0.05 ppm). Moreover, in both the cases, also the two CH_2_(3) chemical shifts proceed parallelly: An enhancement in CH(2) chemical shift results in a quite strong decrease of both two CH_2_(3) ppm values. In benzodioxane amide **(*R*,*S*′)‐Ia**, H_cis_ on C(3) is 0.05 ppm lower than the same H_cis_ on C(3) of amide **(*S*,*S*′)‐Ia**. A similar difference can be noticed when comparing the two benzoxathiane‐2‐carboxamides **6**: The most polar amide shows both the higher CH(2) chemical shift (4.77 ppm) and the lower CH_2_(3) chemical shifts (3.38 and 3.17 ppm of H_cis_ and H_trans_, respectively). Moreover, no differences between the (*S*)‐PEA CH ppm values of the two related diastereoisomers could be detected, neither in the benzodioxane‐2‐carboxamides nor in the benzoxathiane‐2‐carboxamides **6**. This nice progression supported the structural analogy around the chiral center.

Even when considering the benzodioxane‐3‐carboxamides **10** (Figure 4C), we can observe a relevant difference between the two CH(3) values (0.04 ppm), showing a higher chemical shift for the polar amide. In this case, there is not a concomitant decrease of the two CH_2_(2) chemical shifts, which revealed to be more similar.

Furthermore, when considering the chemical shifts of the (*S*)‐PEA hydrogens, two different considerations could be done. On one hand, the (*S*)‐PEA CH ppm values are identical for both the benzodioxane amides and the 2‐substituted benzoxathiane ones. A visible difference could be indeed interestingly noticed between the two (*S*)‐PEA CH chemical shifts of the 3‐substituted benzoxathiane carboxamides. Moreover, this difference results also in the determination of two different coupling constants: the one between (*S*)‐PEA CH and CH_3_ remains fixed (7.0 Hz), whereas the one between (*S*)‐PEA CH and NH differs for the two diastereoisomers (6.98 or 7.3 Hz). Finally, a strong difference in ppm values of (*S*)‐PEA CH_3_ is always present and with a common trend. For all the three couples of amides, the less polar amide is the one having the lower (*S*)‐PEA CH_3_ chemical shift.

Furthermore, while comparing the melting points of the different amides couples (Table 1), a comparable trend can be observed: The most polar amide of each couple is also the one having the higher melting point. This stable tendency further suggests a structural analogy of the chiral center, among benzodioxane and benzoxathiane derivatives.

Finally, a parallel direction could be also noticed when considering the optical rotations of all the amides, as reported in Tables 1 and 3. The 3‐regioisomer gets out of this trend and may be due to the strong contribution of sulfur atom on the polarity of the chiral center, which could strongly affect the optical rotation, causing, for both the amides, an inversion in the [α]_*D*_^25^ values. The tendency observed for all the evaluated physical–chemical data moved us in hypothesizing, which was the absolute configuration of the novel amides **6** and **10** and, as a result, also the ones of the relative carboxylic acids.

**TABLE 3:** Optical rotations of benzodioxane and benzoxathiane compounds

Amides	Optical rotation	Acids	Optical rotation
**(*S*,*S*′)‐Ia**	−41.4	**(*S*)‐I**	−63.8^29^
**(*R*,*S*′)‐Ia**	+71.8	**(*R*)‐I**	+63.0^29^
**(*R*,*S*′)‐6**	−61.2	**(*R*)‐1**	−100.3
**(*S*,*S*′)‐6**	+95.7	**(*S*)‐1**	+100.22

The hydrolysis of the amides, for the 2‐substituted benzoxathianes, and the relative transformation into the carboxylic acids were achieved avoiding any racemization. The accomplished carboxylic acids **1** were then fully characterized, by using NMR, chiral HPLC, and polarimetry.

When comparing the optical rotation values of benzodioxane and 2‐substitued benzoxathiane carboxylic acids (see Table 3), it could be noticed how both the acids coming from the former eluted amides show a negative alpha value, whereas the latter eluted is positive, in both the cases. This similar outcome, both in carboxamide and in carboxylic acids, further confirm the analogous spatial disposition of the C(2). Nevertheless, the presence of the sulfur atom results in the different definition of the chiral center, as Cahn–Ingold–Prelog priority rules assess.

Despite the scarcity of literature data on benzoxathiane derivatives, García‐Rubiño and coworkers^4^ enantioselective synthesized both (*S*)‐ and (*R*)‐2,3‐dihydro‐1,4‐benzoxathiin‐2‐methanol, starting from chirally defined epichlorohydrin. They reported both the absolute configuration and the relative optical rotations.

As a result, to further confirm the supposed absolute configurations of acids **1**, we decided to reduce **(*R*)‐1** carboxylic acid with LiAlH_4_, following reduction conditions used on both racemic^30^ and chiral^29^ benzodioxane, thus achieving the known hydroxymethyl compound^4^, ^5^ (full experimental conditions are reported in the relative section). After purification, we evaluate the [α]_*D*_^25^ values and polarimetry data completely confirmed the supposed chirality.

Concerning 3‐substituted derivatives, we previously observed the chiral instability of benzodioxane derivatives^31^ when treated in basic conditions. Here, we were also aware of benzoxathiane scaffold 3‐substituted chemical instability in bases, considering what saw when treating the racemic ethyl ester in pure (*S*)‐PEA. As a result, we exclude to obtain the corresponding acids by using any strong organic or inorganic bases.

Nevertheless, the chiral instability of the same scaffold in acids completely astonished us. We indeed followed the same synthetic procedure we developed for the obtainment of 1,4‐benzodioxane carboxylic acids,^31^ which resulted to be productive for the 2‐regioisomer.

When operating in the same conditions for the 3‐regioisomer no degradation of the moiety occurred, as observed in base, but the racemization on the chiral center was complete. Neither changes in temperatures nor in acidic potencies were able to limit the racemization.

We also tried to hydrolyze the amide bond in a completely different manner. We followed the procedure previously developed for the hydrolysis of the lateral chain of several penicillins. We firstly formed the imidoyl chloride derivative, by treating the amide with PCl_5_ at low temperatures in DCM; we further transformed it into the imino ether, by adding absolute ethanol to the reaction mixture, and we finally hydrolyzed the carboximidate in acid and cold conditions, thus achieving the ethyl carboxylate of 2. Unfortunately, also operating with these reaction conditions, we obtained the fully racemic ester.

## 4 CONCLUSION

Concluding, with this work, we developed a robust and defined method for the preparation of *S*‐phenylethyl‐1,4‐benzoxathiane‐2‐ and 3‐ carboxamides. *S*‐phenylethylamine resulted to be a useful enantiopure chiral auxiliary for achieving amides showing high enantiomeric excesses and that could be easily purified on silica gel by flash chromatography. The four isolated amides were fully characterized, and their absolute configuration was firstly hypothesized, by comparison with known benzodioxane ones, and then further confirmed, by transformation into known chiral 1,4‐benzoxathiane‐2‐substituted.

These enantiopure amides could be good intermediates for the achievement of several enantiopure 2‐ and 3‐benzoxathiane derivatives.

Here, we decided to transform the 2‐substituted amides into their corresponding acids, which were quantitatively achieved and isolated. Their physical chemical properties, as well as their absolute configuration, were calculated and defined.

Unfortunately, we achieved the 1,4‐benzoxathiane‐3‐carboxylic acids only as racemates. Nevertheless, we are going on working on their isolation as enantiopure compounds, by developing an enantioselective synthesis or by using a different resolution method, which should avoid any acid or basic conditions.

## Supporting information


**Data S1.** Supporting Information

## Data Availability

The data that support the findings of this study are available from the corresponding author upon reasonable request.
