# Supplementary material for: Resolution via diastereomeric amides of enantiopure 1,4‐benzoxathian‐2‐ and 3‐carboxylic acids and determination of their configuration
Source: Chirality. 2022 May 20;34(8):1053–64. doi: 10.1002/chir.23474 (PMC9541051; doi:10.1002/chir.23474)
Supplement: Supplementary file 1 — Data S1. Supporting Information [file CHIR-34-1053-s001.pdf]

Supporting Information:

"Resolution via diastereomeric amides of enantiopure 1,4-benzoxathian-2- and 3- carboxylic acids and determination of their configuration"

Summary

Ethyl 2,3-dibromopropionate (3) 3
Ethyl 2-bromoacrylate (4) 4
Ethyl 1,4-benzoxathian-2-carboxylate (5) 5
(2*S*)-N-((*S*)-1-phenylethyl)-2,3-dihydrobenzo-(1,4)-oxathiine-2-carboxamide (*S,S'*-6) 6
(2*R*)-N-((*S*)-1-phenylethyl)-2,3-dihydrobenzo-(1,4)-oxathiine-2-carboxamide (*R,S'*-6) 7
(2*S*) -2,3-dihydrobenzo-(1,4)-oxathiine-2-carboxylic acid (*S*-1) 8
(2*R*) -2,3-dihydrobenzo-(1,4)-oxathiine-2-carboxylic acid (*R*-1) 8
Acryloyl chloride (7) 9
(*S*)-N-(1-phenylethyl)acrylamide (8) 10
2,3-dibromo-N-((S)-1-phenylethyl)propanamide (9) 11
Supposed (3*R*)-N-((*S*)-1-phenylethyl)-2,3-dihydrobenzo-(1,4)-oxathiine-3-carboxamide (*R,S'*-10) 12
Supposed (3*S*)-N-((*S*)-1-phenylethyl)-2,3-dihydrobenzo-(1,4)-oxathiine-3-carboxamide (*S,S'*-10) 13
2,3-dihydrobenzo-(1,4)-oxathiine-3-carboxylic acid (*rac*-2) 14
(*S*)-N-((*S*)-1-phenylethyl)-2,3-dihydrobenzodioxine-2-carboxamide (*S,S'*)-Ia 15
(*R*)-N-((*S*)-1-phenylethyl)-2,3-dihydrobenzodioxine-2-carboxamide (*R,S'*)-Ia 16
HPLC methods 17
METHOD A: 17
Ethyl 1,4-benzoxathian-2-carboxylate (5) 17
METHOD B: 17
(2*R*)-*N*-((*S*)-1-phenylethyl)-2,3-dihydrobenzo-(1,4)-oxathiine-2-carboxamide, (2*S*)-*N*-((*S*)-1-phenylethyl)-2,3-dihydrobenzo-(1,4)-oxathiine-2-carboxamide, (2*R*)-*N*-((*S*)-1-phenylethyl)-2,3-dihydrobenzo-(1,4)-oxathiine-3-carboxamide and (2*S*)-*N*-((*S*)-1-phenylethyl)-2,3-dihydrobenzo-(1,4)-oxathiine-3-carboxamide (6 and 10) 17
METHOD C: 17
(2*S*) and (2*R*)-2,3-dihydrobenzo-(1,4)-oxathiine-2-carboxylic acid (*S*/*R*-1) 17
METHOD D: 17
(3*S*) and (3*R*)-2,3-dihydrobenzo-(1,4)-oxathiine-2-carboxylic acid (*S*/*R*-2) 17
HPLC chromatograms 18
Ethyl 1,4-benzoxathian-2-carboxylate (5) 18

(2*R*)-*N*-((*S*)-1-phenylethyl)-2,3-dihydrobenzo-(1,4)-oxathiine-2-carboxamide (*R*,*S'*-6) ... 19
(2*S*)-*N*-((*S*)-1-phenylethyl)-2,3-dihydrobenzo-(1,4)-oxathiine-2-carboxamide (*S*,*S'*-6) ... 19
(2*S*)-2,3-dihydrobenzo-(1,4)-oxathiine-2-carboxylic acid (*S*-1) ... 20
(2*R*)-2,3-dihydrobenzo-(1,4)-oxathiine-2-carboxylic acid (*R*-1) ... 20
(2*R*)-*N*-((*S*)-1-phenylethyl)-2,3-dihydrobenzo-(1,4)-oxathiine-3-carboxamide (*R*,*S'*-10) ... 21
(2*S*)-*N*-((*S*)-1-phenylethyl)-2,3-dihydrobenzo-(1,4)-oxathiine-3-carboxamide (*S*,*S'*-10) ... 21
2,3-dihydrobenzo-(1,4)-oxathiine-2-carboxylic acid (*rac*-2) ... 22
DSC ... 23
(2*S*)-*N*-((*S*)-1-phenylethyl)-2,3-dihydrobenzo-(1,4)-oxathiine-2-carboxamide (*S*,*S'*-6) ... 23
(2*S*)-*N*-((*S*)-1-phenylethyl)-2,3-dihydrobenzo-(1,4)-oxathiine-3-carboxamide (*S*,*S'*-10) ... 24
(2*R*)-N-((*S*)-1-phenylethyl)-2,3-dihydrobenzo-(1,4)-oxathiine-3-carboxamide (*R*,*S'*-10) ... 24
(2*S*)-2,3-dihydrobenzo-(1,4)-oxathiine-2-carboxylic acid (*S*-1) and (2*R*)-2,3-dihydrobenzo-(1,4)-oxathiine-2-carboxylic acid (*R*-1) ... 25
2,3-dihydrobenzo-(1,4)-oxathiine-2-carboxylic acid (*rac*-2) ... 26
HIGH-RESOLUTION MASS ANALYSIS ... 27
(2*S*)-N-((*S*)-1-phenylethyl)-2,3-dihydrobenzo-(1,4)-oxathiine-2-carboxamide (*S*,*S'*-6) ... 27
(2*R*)-*N*-((*S*)-1-phenylethyl)-2,3-dihydrobenzo-(1,4)-oxathiine-2-carboxamide (*R*,*S'*-6) ... 28
(2*S*)-2,3-dihydrobenzo-(1,4)-oxathiine-2-carboxylic acid (*S*-1) ... 29
(2*R*)-2,3-dihydrobenzo-(1,4)-oxathiine-2-carboxylic acid (*R*-1) ... 30
(2*S*)-*N*-((*S*)-1-phenylethyl)-2,3-dihydrobenzo-(1,4)-oxathiine-3-carboxamide (*S*,*S'*-10) ... 31
(2*R*)-*N*-((*S*)-1-phenylethyl)-2,3-dihydrobenzo-(1,4)-oxathiine-3-carboxamide (*R*,*S'*-10) ... 32
2,3-dihydrobenzo-(1,4)-oxathiine-2-carboxylic acid (*rac*-2) ... 33

Ethyl 2,3-dibromopropionate (3)

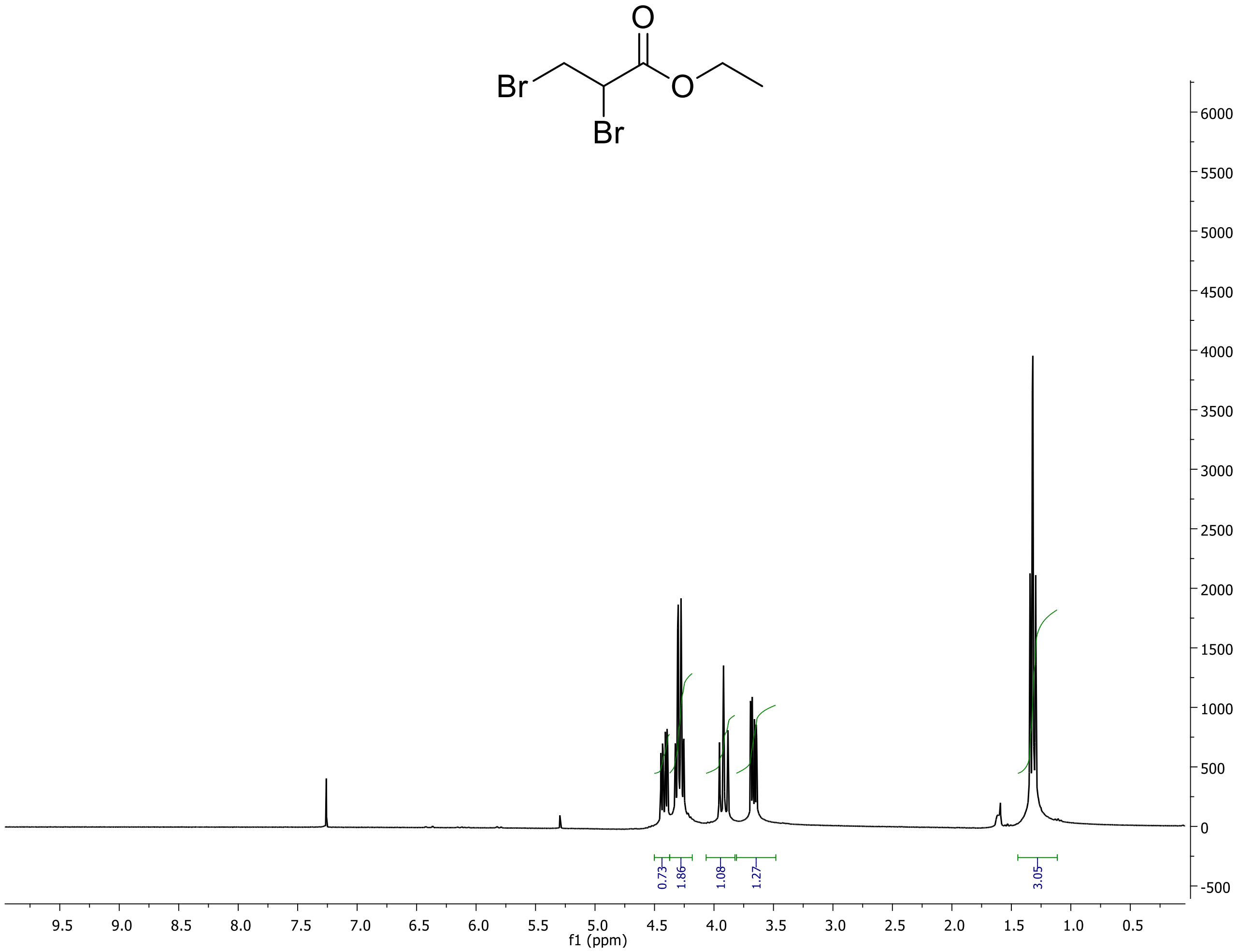

^{1}H NMR was performed in $CDCl_3$ at 300 MHz.

Ethyl 2-bromoacrylate (4)

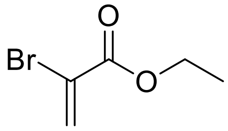

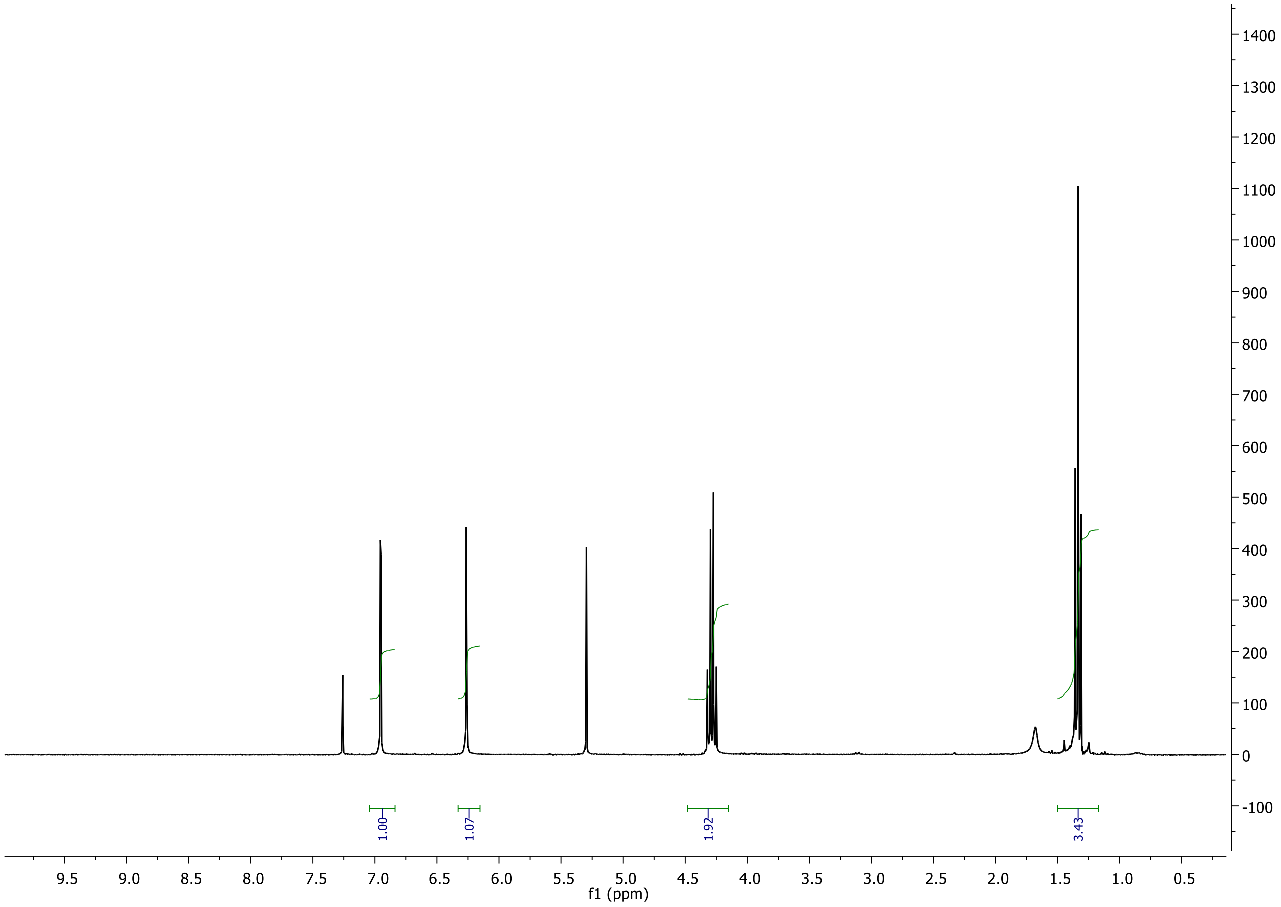

^{1}H NMR was performed in $CDCl_3$ at 300 MHz.

Ethyl 1,4-benzoxathian-2-carboxylate (5)

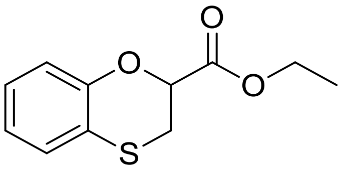

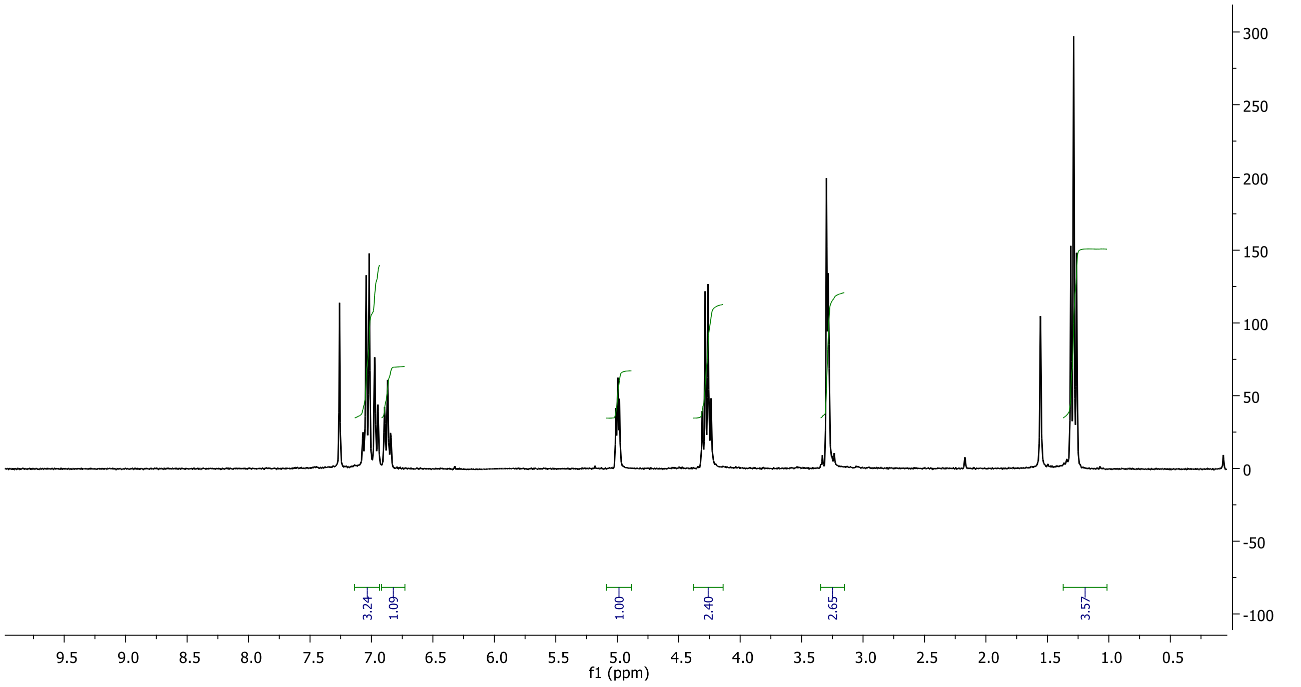

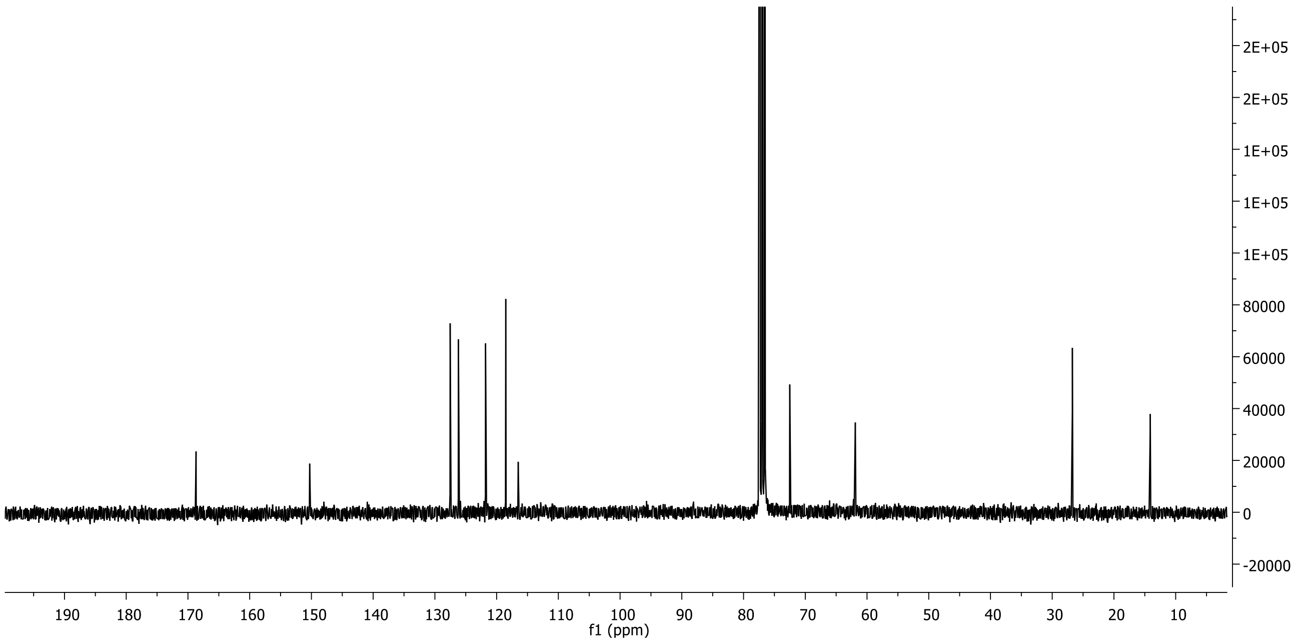

^{1}H- and ^{13}C- NMR were performed in $CDCl_3$ at 300 and 75 MHz, respectively.

(2*S*)-N-((*S*)-1-phenylethyl)-2,3-dihydrobenzo-(1,4)-oxathiine-2-carboxamide (*S,S'*-6)

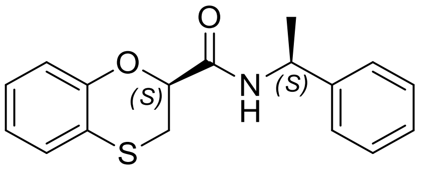

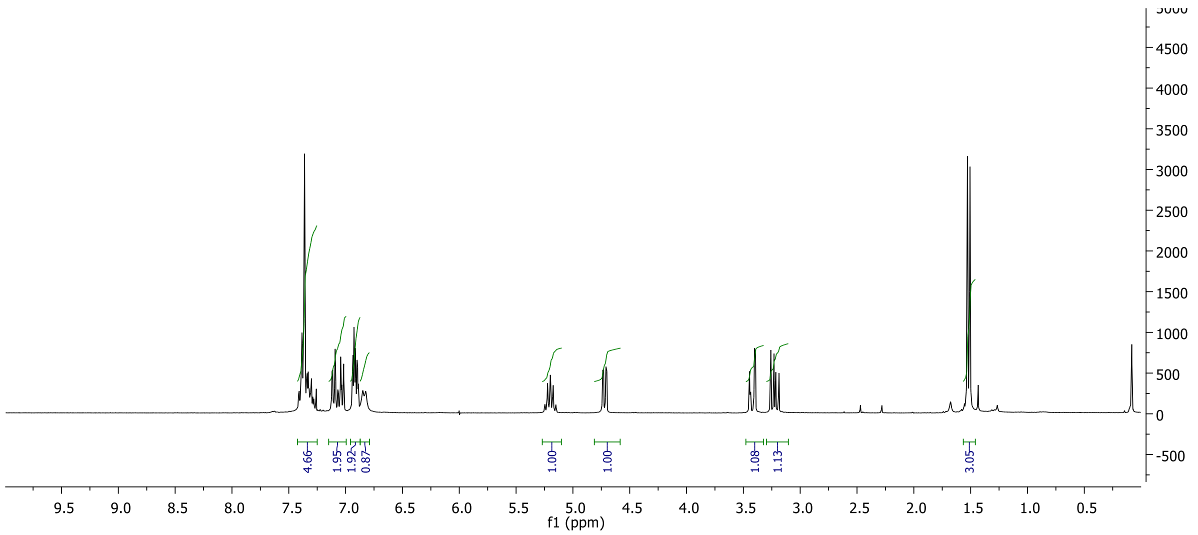

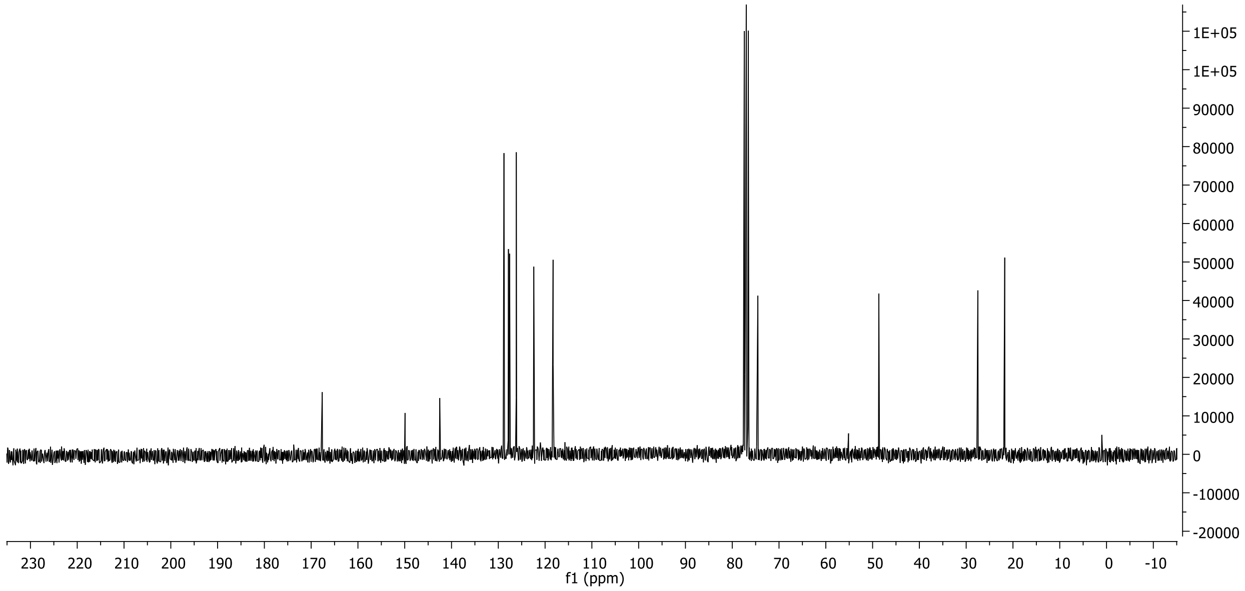

^{1}H- and ^{13}C- NMR were performed in $CDCl_3$ at 300 and 75 MHz, respectively.

(2*R*)-N-((*S*)-1-phenylethyl)-2,3-dihydrobenzo-(1,4)-oxathiine-2-carboxamide (*R,S'*-6)

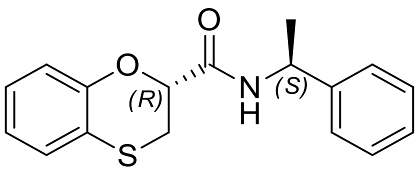

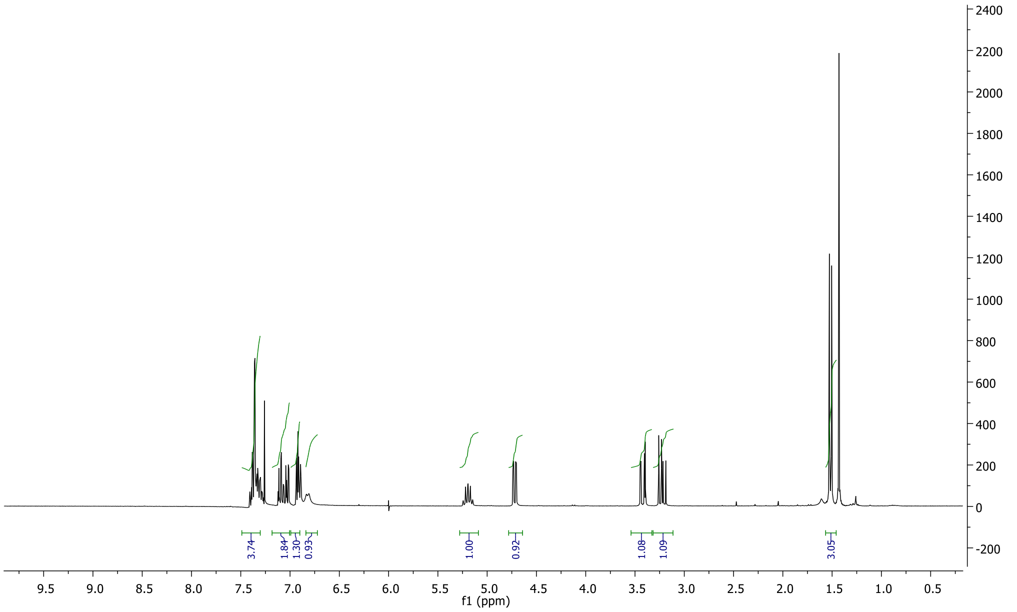

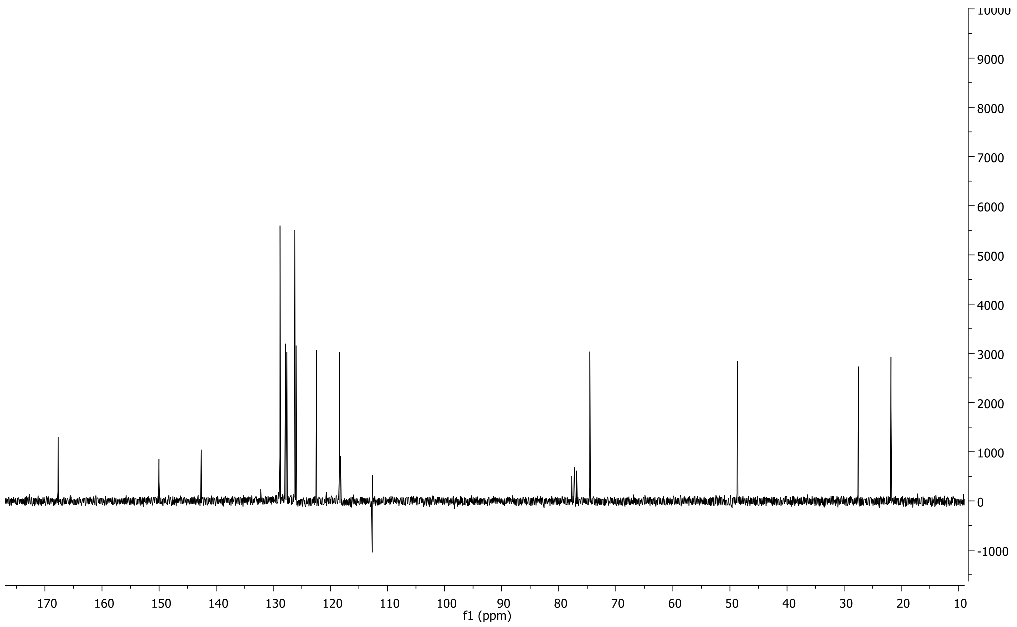

^{1}H- and ^{13}C- NMR were performed in $CDCl_3$ at 300 and 75 MHz, respectively.

(2*S*) -2,3-dihydrobenzo-(1,4)-oxathiine-2-carboxylic acid (*S*-1)

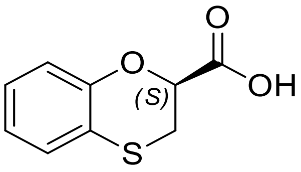

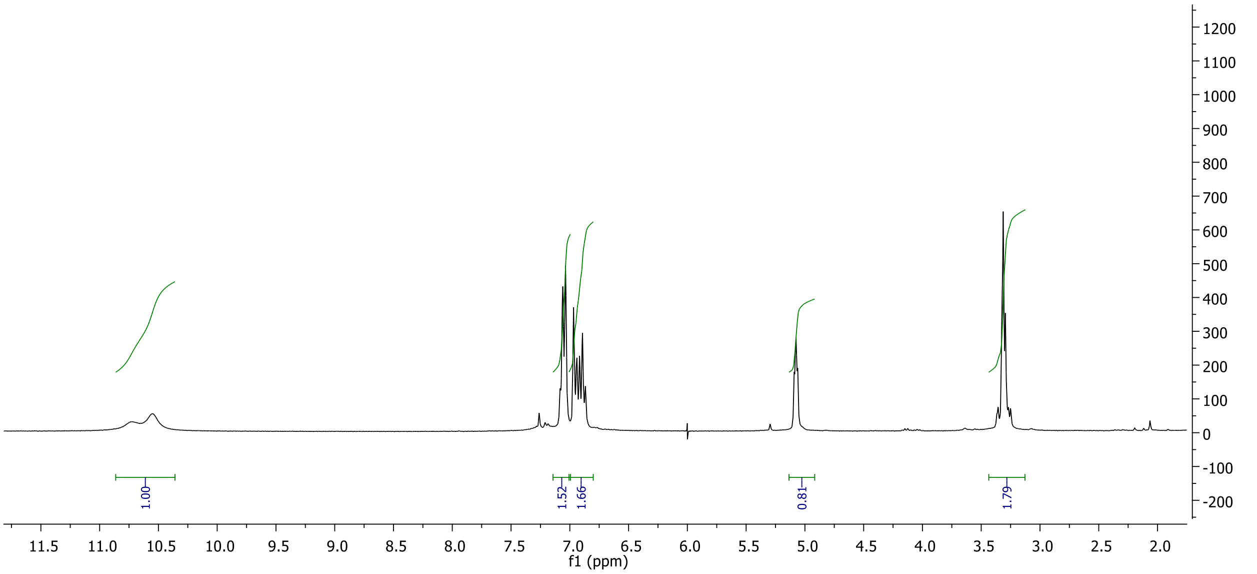

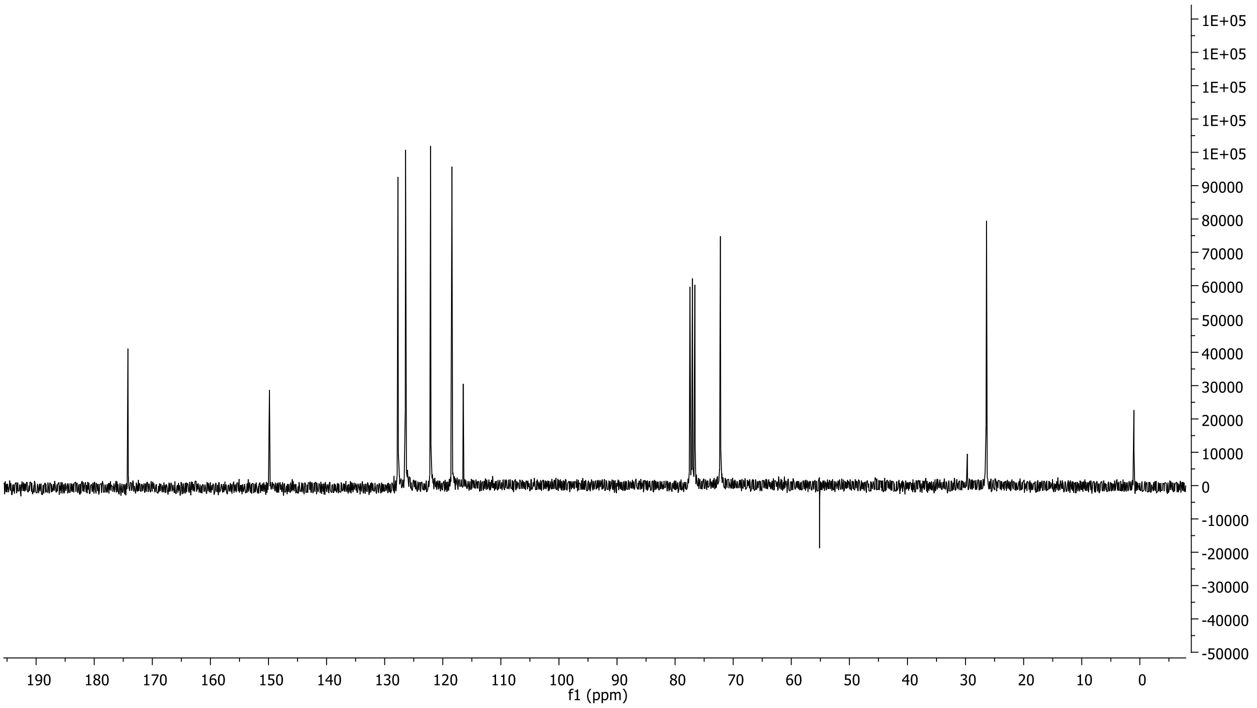

^{1}H- and ^{13}C- NMR were performed in $CDCl_3$ at 300 and 75 MHz, respectively.

(2*R*) -2,3-dihydrobenzo-(1,4)-oxathiine-2-carboxylic acid (*R*-1)

Both ^{1}H- and ^{13}-C NMR spectra are identical to that of (S)-1 enantiomer, here above reported.

Acryloyl chloride (7)

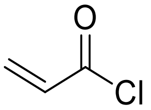

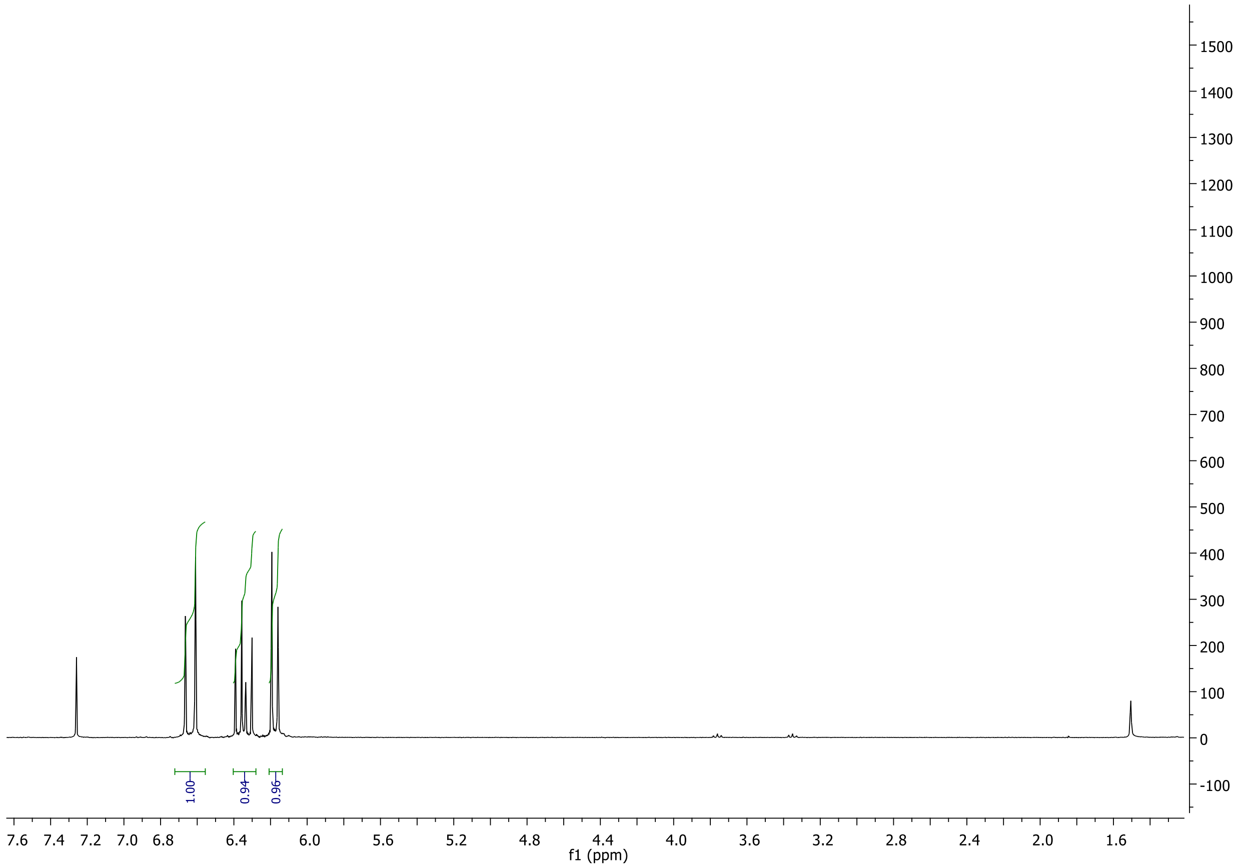

^{1}H NMR was performed in $CDCl_3$ at 300 MHz.

(*S*)-N-(1-phenylethyl)acrylamide (8)

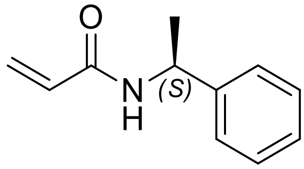

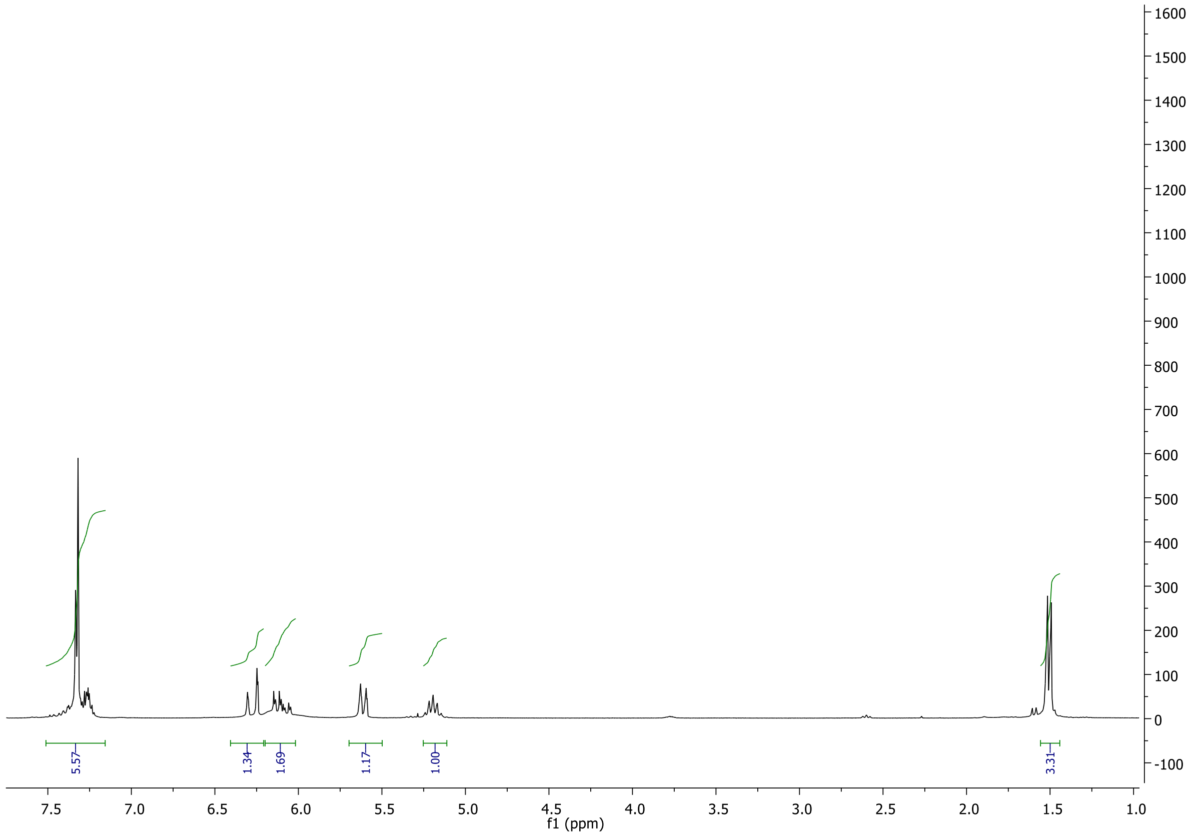

^{1}H NMR was performed in $CDCl_3$ at 300 MHz.

2,3-dibromo-N-((S)-1-phenylethyl)propanamide (9)

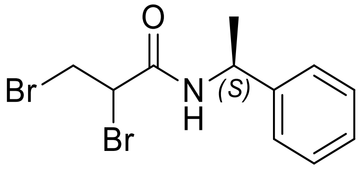

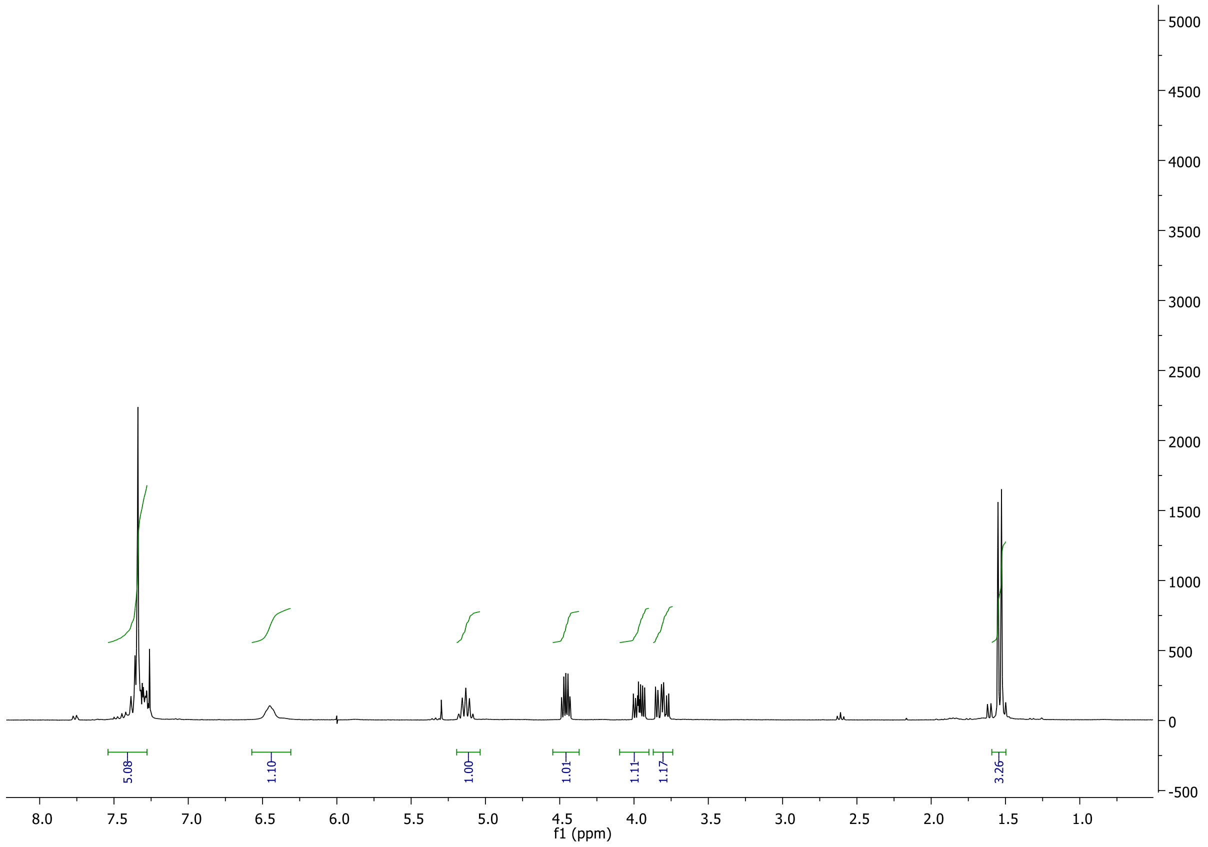

^{1}H NMR was performed in $CDCl_3$ at 300 MHz.

Supposed (3*R*)-N-((*S*)-1-phenylethyl)-2,3-dihydrobenzo-(1,4)-oxathiine-3-carboxamide (*R*,*S'*-10)

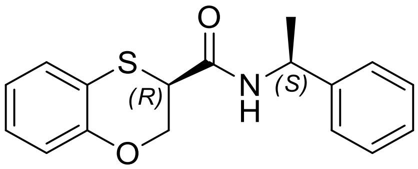

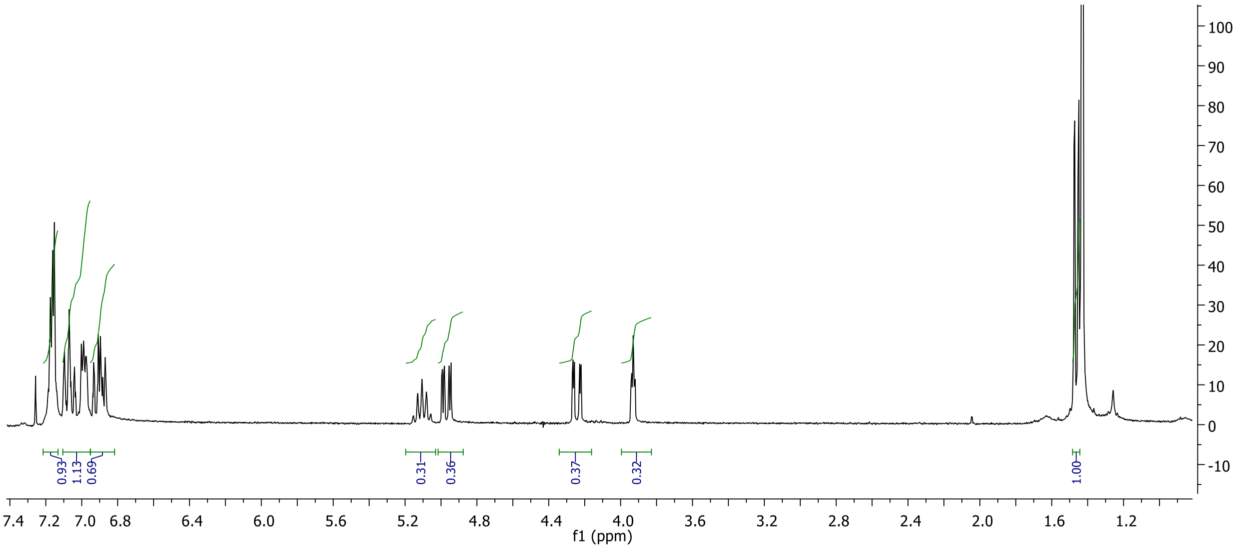

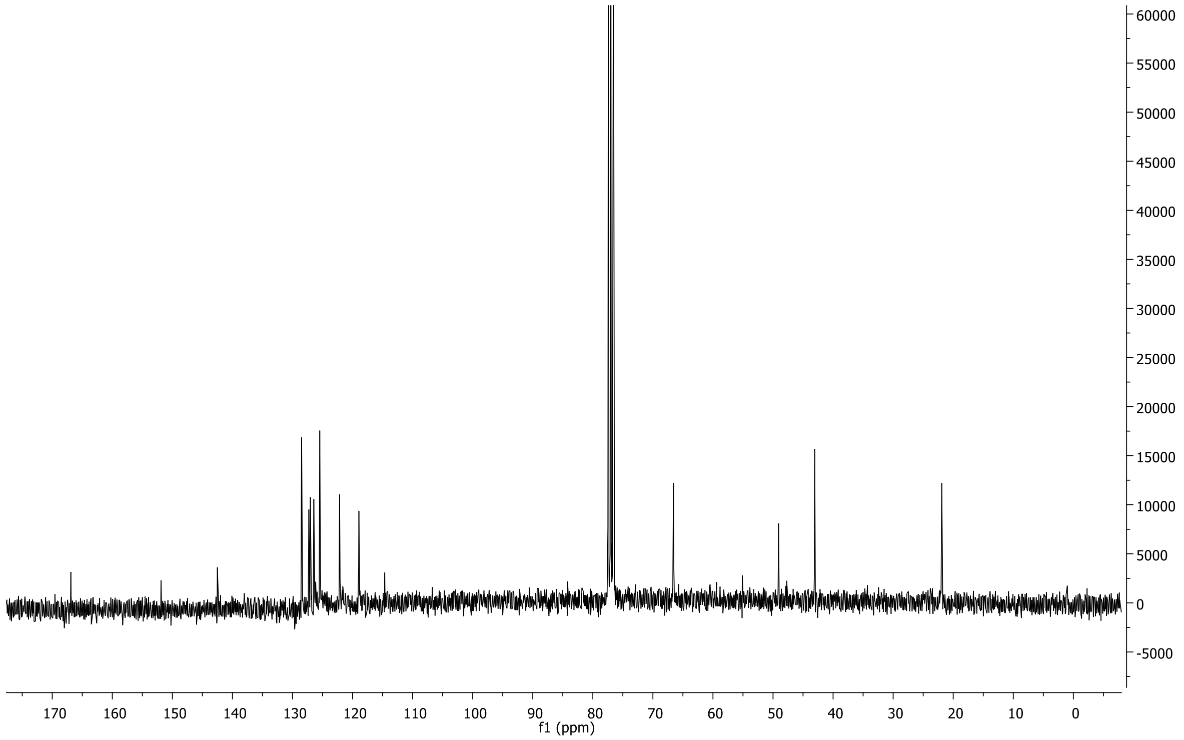

^{1}H- and ^{13}C- NMR were performed in $CDCl_3$ at 300 and 75 MHz, respectively.

Supposed (3*S*)-N-((*S*)-1-phenylethyl)-2,3-dihydrobenzo-(1,4)-oxathiine-3-carboxamide (*S,S'*-10)

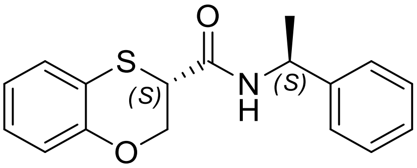

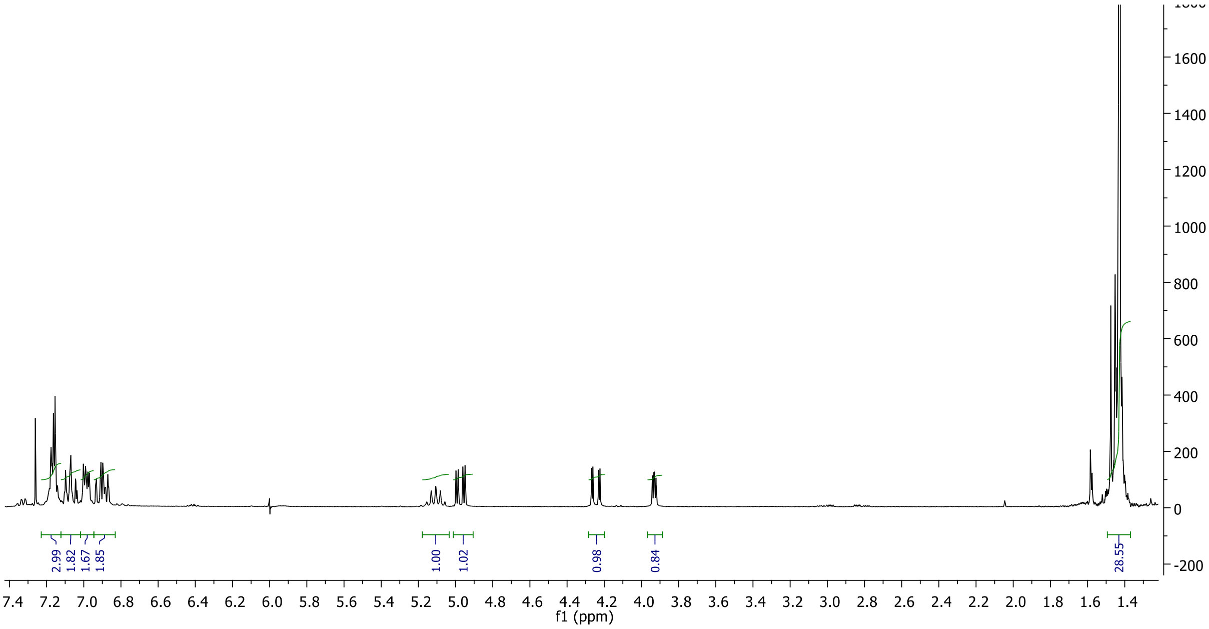

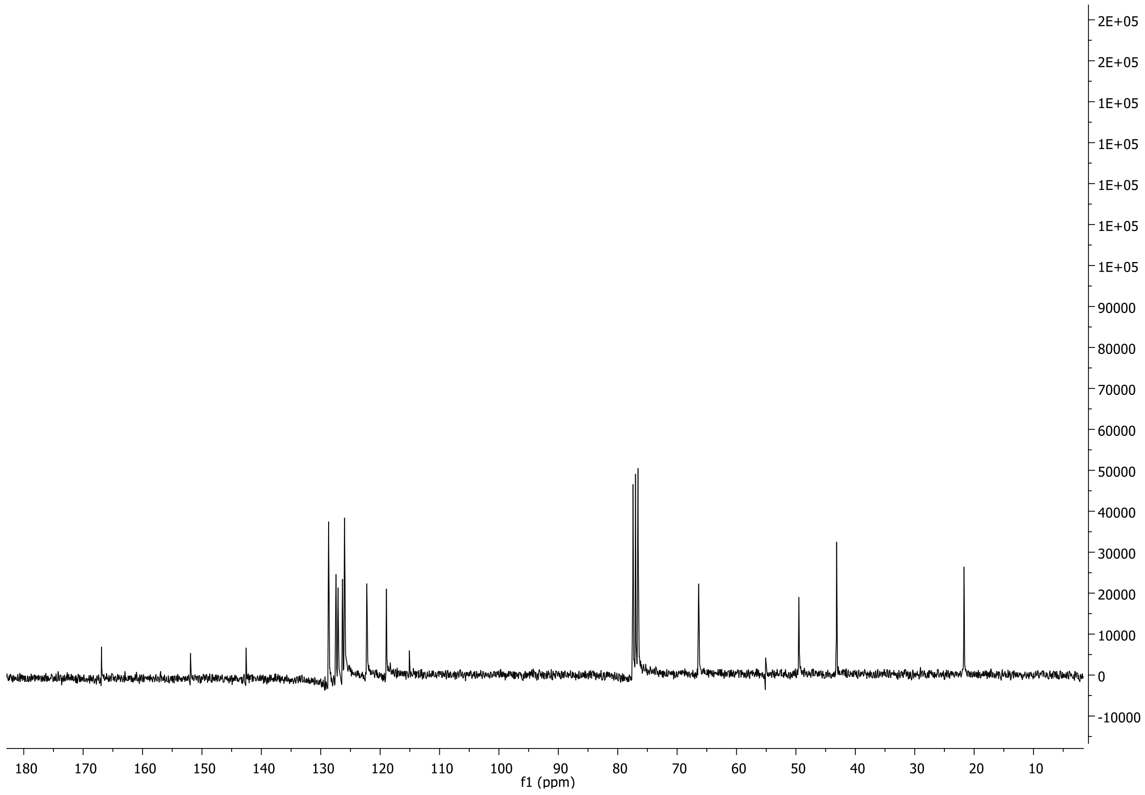

^{1}H- and ^{13}C- NMR were performed in $CDCl_3$ at 300 and 75 MHz, respectively.

2,3-dihydrobenzo-(1,4)-oxathiine-3-carboxylic acid (*rac*-2)

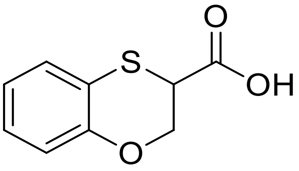

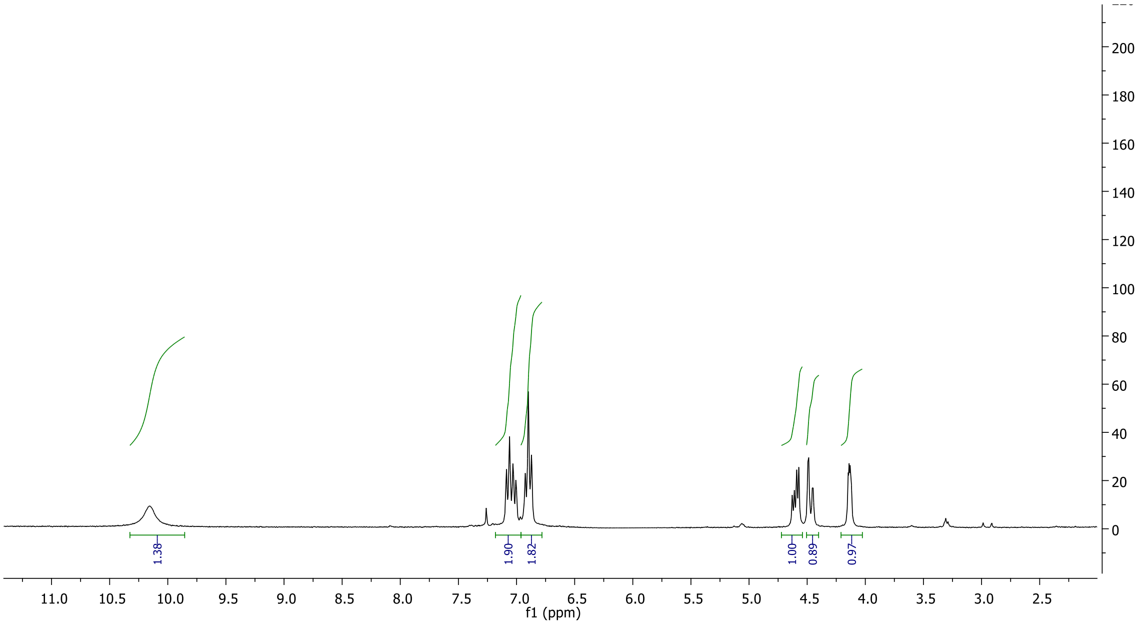

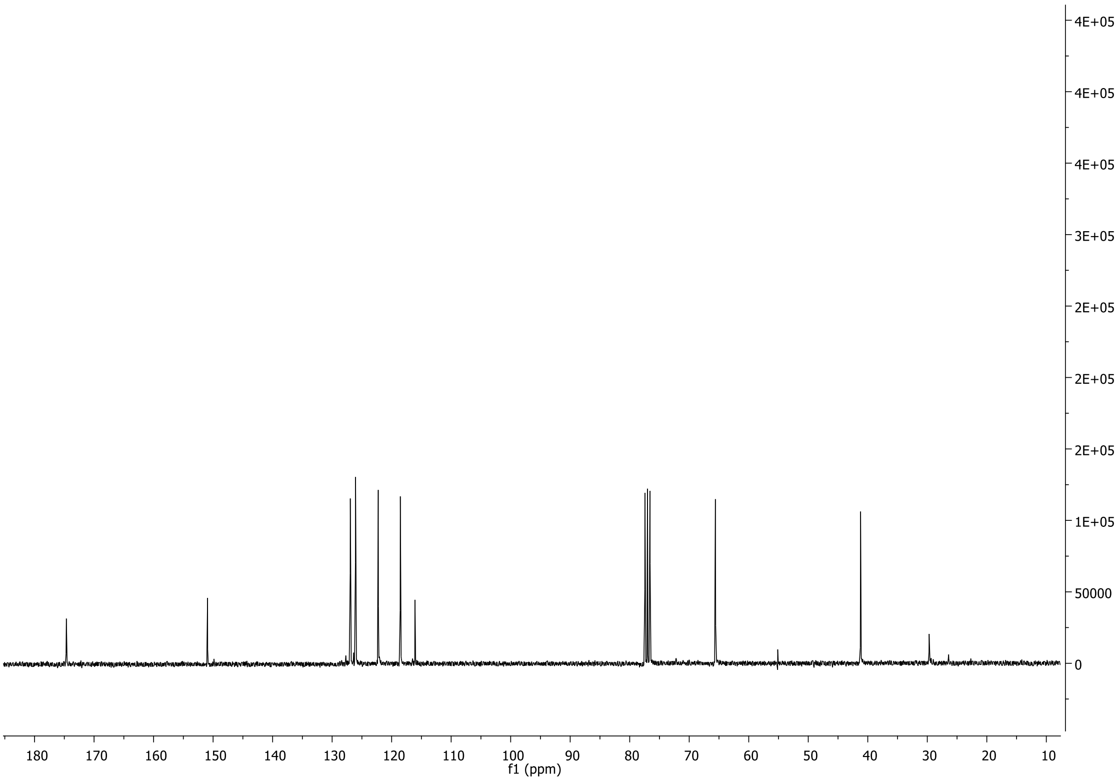

^{1}H- and ^{13}C- NMR were performed in $CDCl_3$ at 300 and 75 MHz, respectively.

(*S*)-N-((*S*)-1-phenylethyl)-2,3-dihydrobenzodioxine-2-carboxamide (*S*,*S'*)-Ia

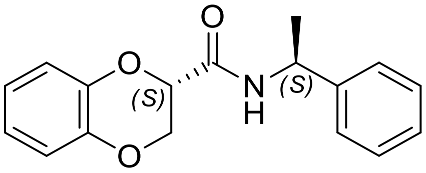

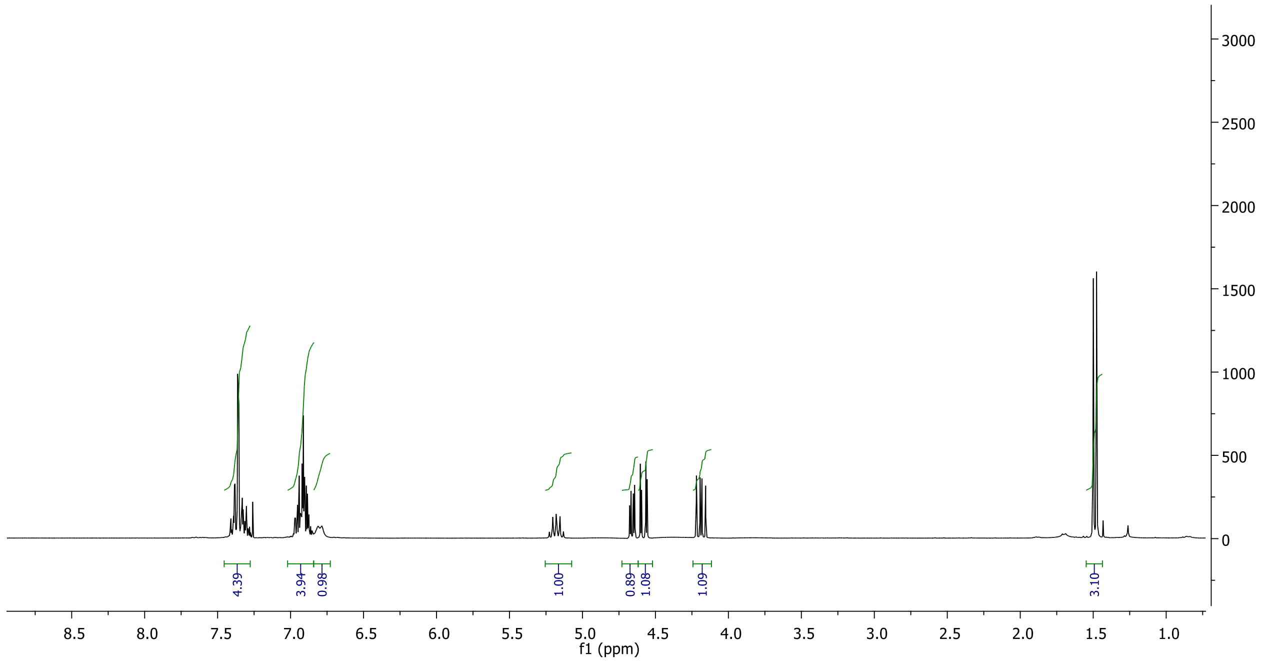

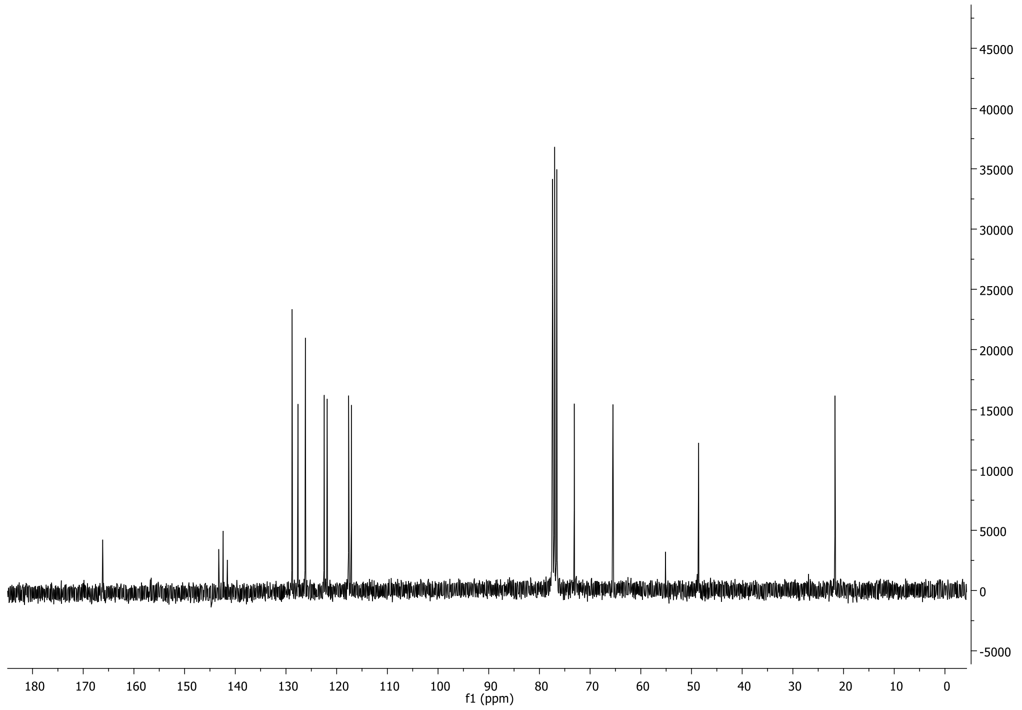

^{1}H- and ^{13}C- NMR were performed in $CDCl_3$ at 300 and 75 MHz, respectively.

(R)-N-((S)-1-phenylethyl)-2,3-dihydrobenzodioxine-2-carboxamide (R,S')-Ia

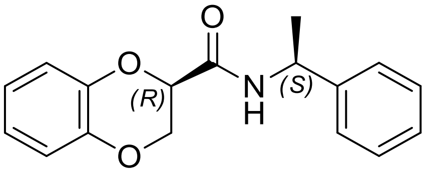

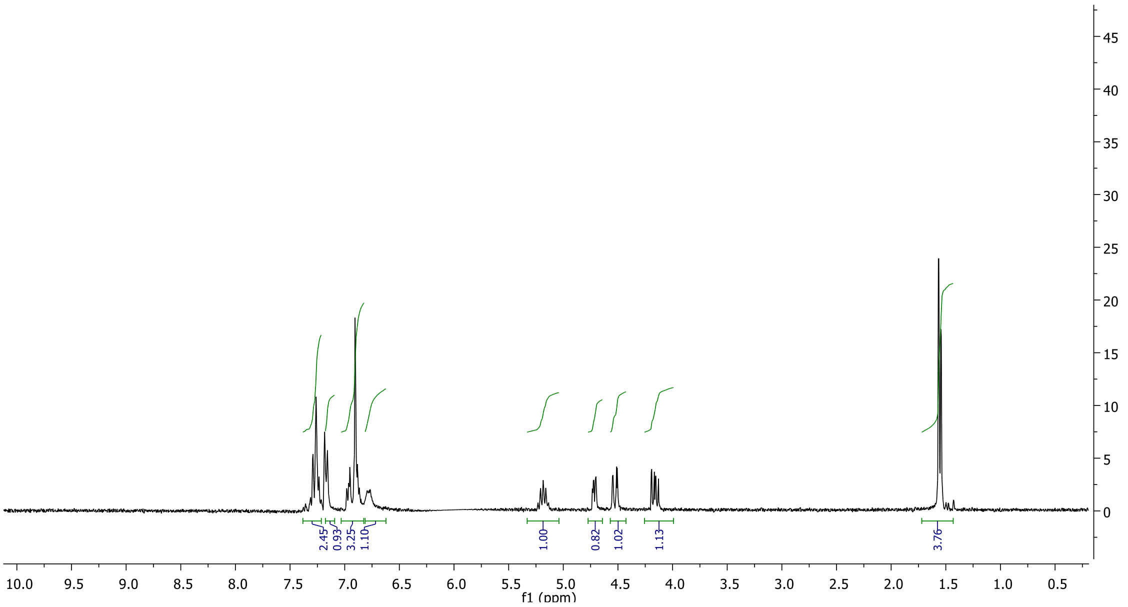

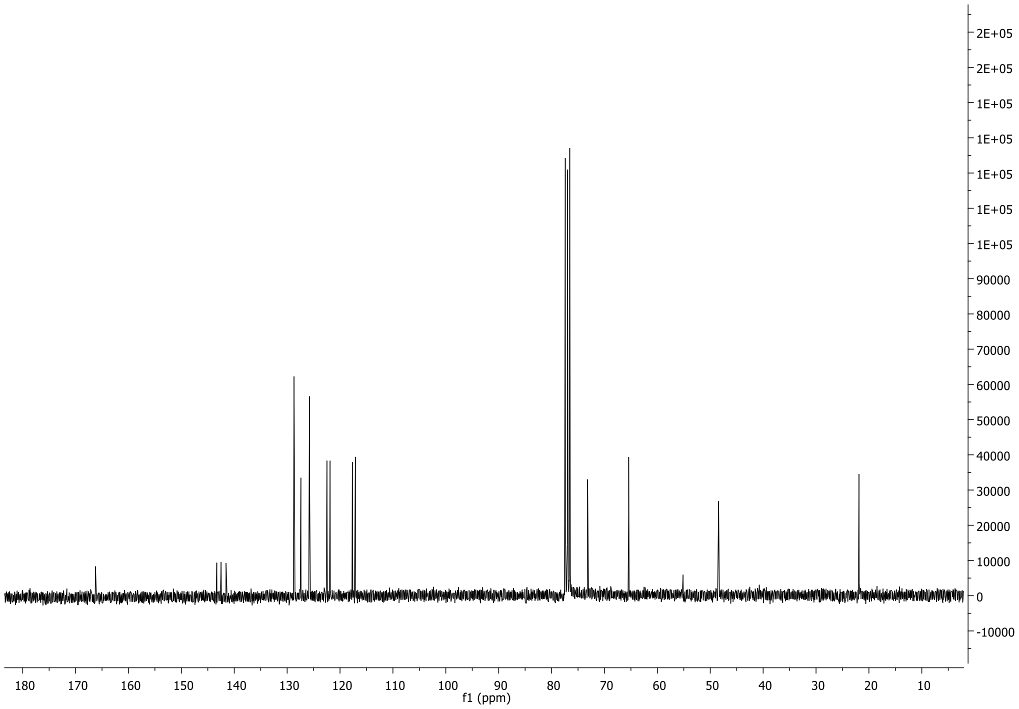

^{1}H- and ^{13}C- NMR were performed in $CDCl_3$ at 300 and 75 MHz, respectively.

HPLC methods

METHOD A:

Ethyl 1,4-benzoxathian-2-carboxylate (5)

Time (minutes)	Solvents %		Flow rate (mL/min)
	ACN	Acetate Buffer pH 4.7	
0	35%	65%	1
10	35%	65%	1
10.5	35%	65%	1
40	35%	65%	1

METHOD B:

(*2R*)-*N*-((*S*)-1-phenylethyl)-2,3-dihydrobenzo-(1,4)-oxathiine-2-carboxamide, (*2S*)-*N*-((*S*)-1-phenylethyl)-2,3-dihydrobenzo-(1,4)-oxathiine-2-carboxamide, (*2R*)-*N*-((*S*)-1-phenylethyl)-2,3-dihydrobenzo-(1,4)-oxathiine-3-carboxamide and (*2S*)-*N*-((*S*)-1-phenylethyl)-2,3-dihydrobenzo-(1,4)-oxathiine-3-carboxamide (6 and 10)

Time (minutes)	Solvents %		Flow rate (mL/min)
	ACN +TFA 0.1%	Water + TFA 0.1%	
0	50%	50%	1
15	50%	50%	1

METHOD C:

(2*S*) and (2*R*)-2,3-dihydrobenzo-(1,4)-oxathiine-2-carboxylic acid (*S*/*R*-1)

Time (minutes)	Solvents %		Flow rate (mL/min)
	Hexane + HCOOH 1.5%	IPA	
0	85%	15%	0.5
30	85%	15%	0.5

METHOD D:

(3*S*) and (3*R*)-2,3-dihydrobenzo-(1,4)-oxathiine-2-carboxylic acid (*S*/*R*-2)

Time (minutes)	Solvents %		Flow rate (mL/min)
	Hexane + HCOOH 1.5%	IPA	
0	90%	10%	0.5
30	90%	10%	0.5

HPLC chromatograms

Ethyl 1,4-benzoxathian-2-carboxylate (5)

T_r= 22.33 minutes, Method A

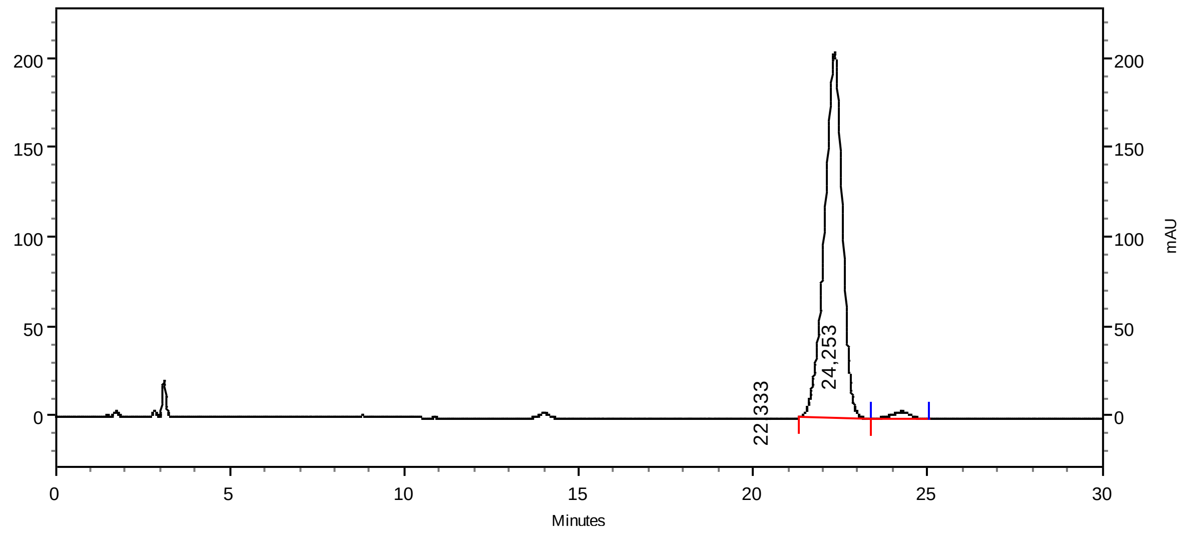

(2*R*)-*N*-((*S*)-1-phenylethyl)-2,3-dihydrobenzo-(1,4)-oxathiine-2-carboxamide (*R*,*S'*-6)

T_r= 7.20 minutes, Method B

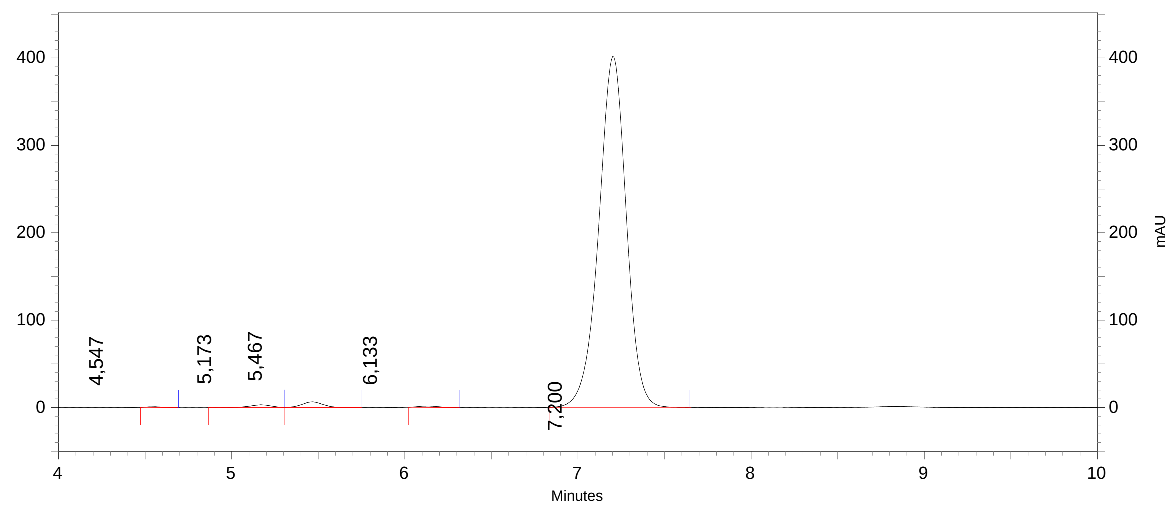

(2*S*)-*N*-((*S*)-1-phenylethyl)-2,3-dihydrobenzo-(1,4)-oxathiine-2-carboxamide (*S*,*S'*-6)

T_r= 6.71 minutes, Method B

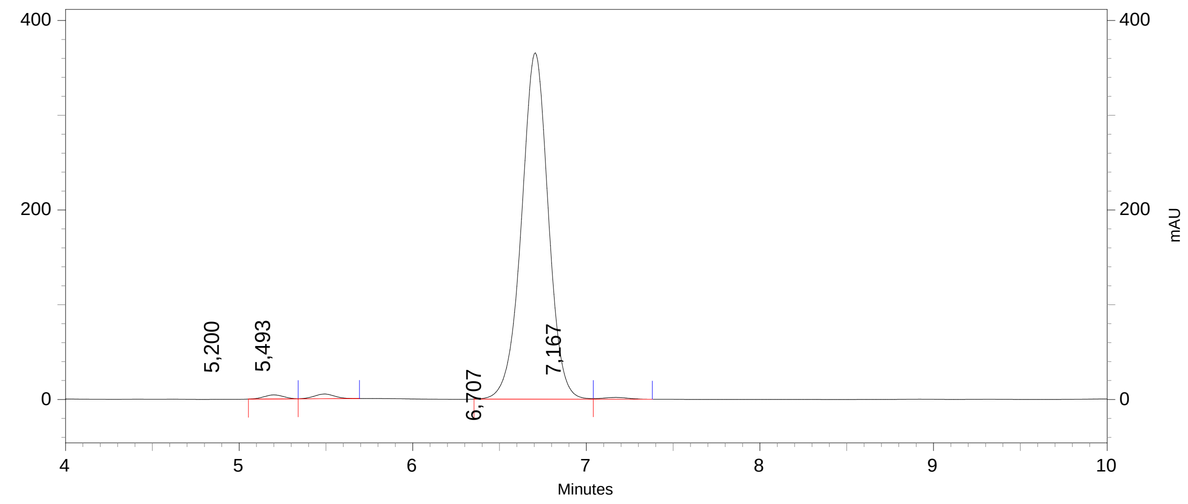

(2*S*)-2,3-dihydrobenzo-(1,4)-oxathiine-2-carboxylic acid (*S*-1)

T_r= 19.7 minutes, Method C

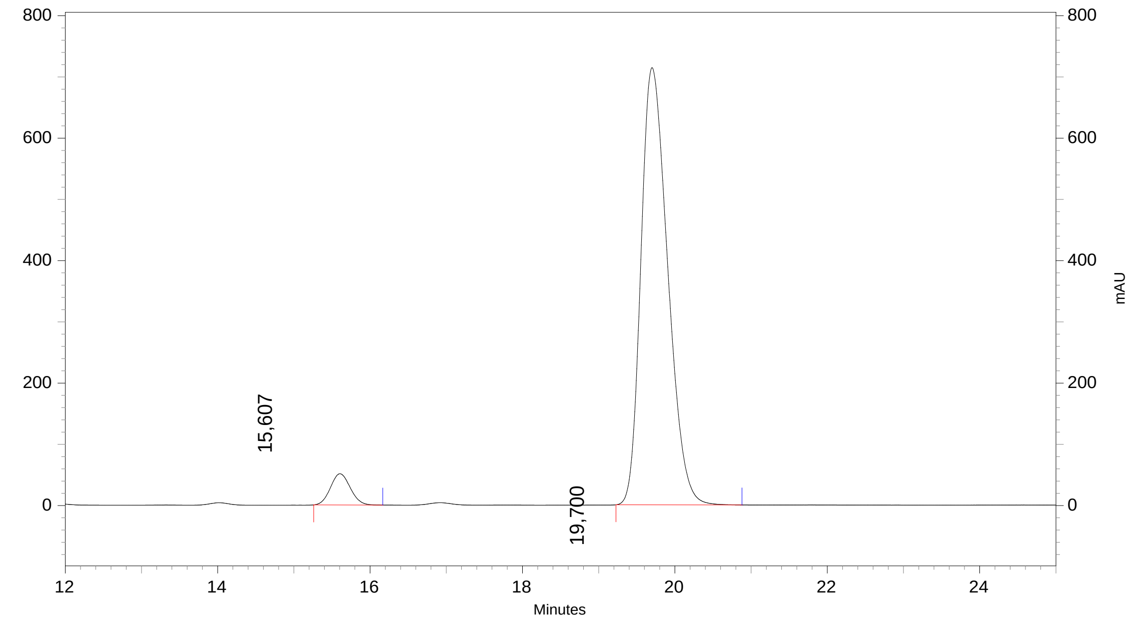

(2*R*)-2,3-dihydrobenzo-(1,4)-oxathiine-2-carboxylic acid (*R*-1)

Tr= 15.43 minutes, Method C

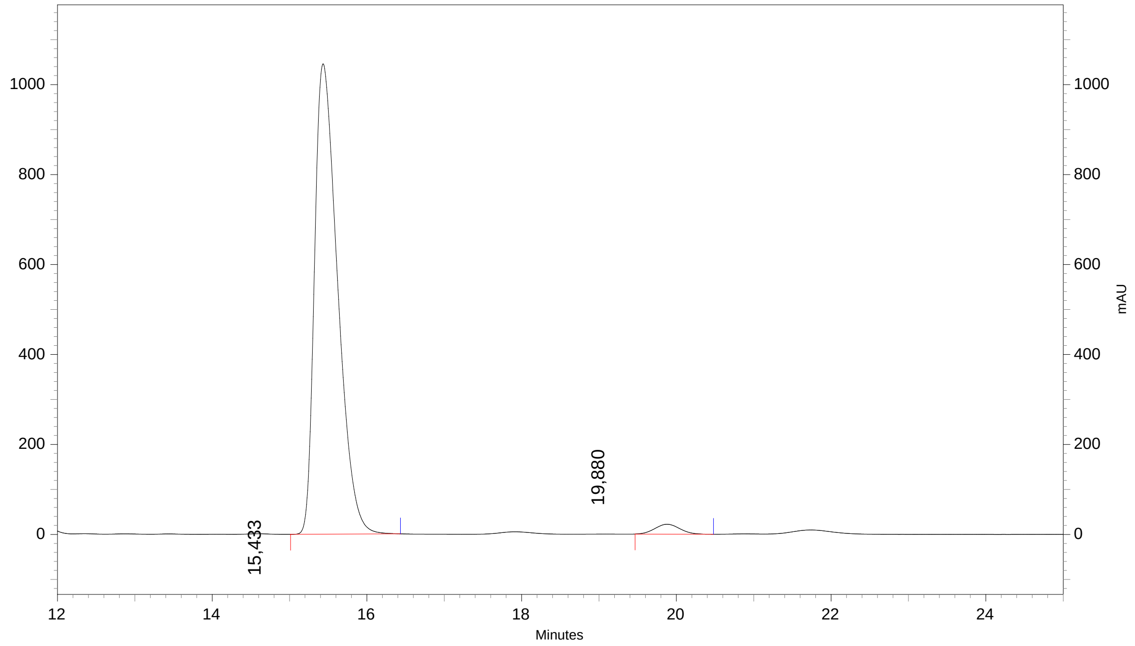

(2*R*)-*N*-((*S*)-1-phenylethyl)-2,3-dihydrobenzo-(1,4)-oxathiine-3-carboxamide (*R*,*S'*-10)

T_r= 6.04 minutes, Method B

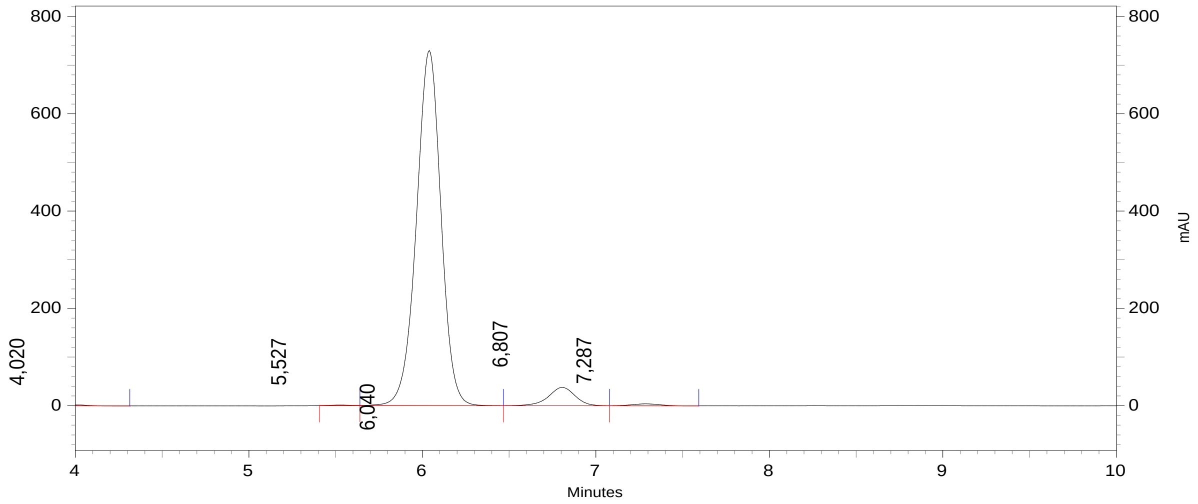

(2*S*)-*N*-((*S*)-1-phenylethyl)-2,3-dihydrobenzo-(1,4)-oxathiine-3-carboxamide (*S*,*S'*-10)

T_r= 5.72 minutes, Method B

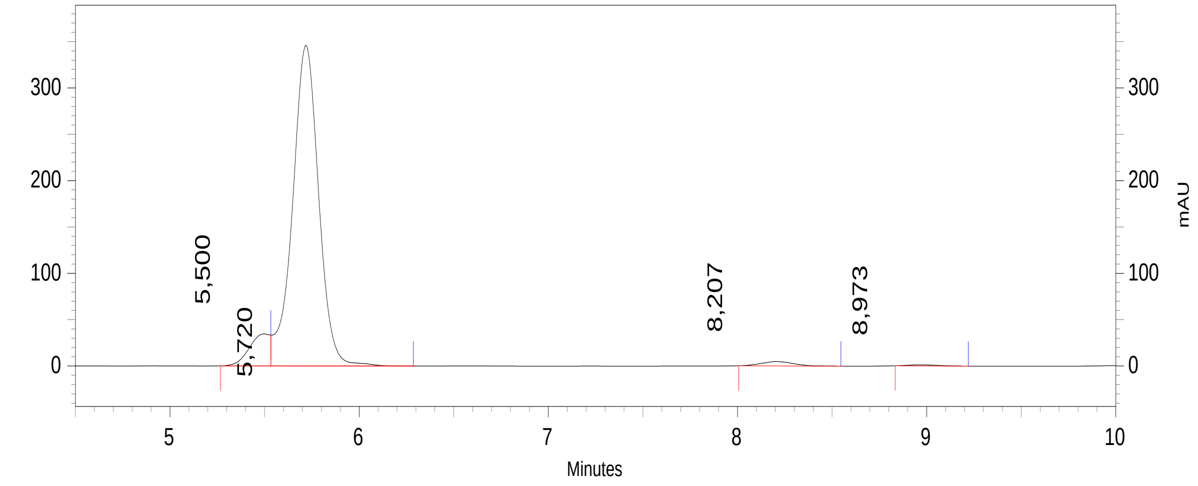

2,3-dihydrobenzo-(1,4)-oxathiine-2-carboxylic acid (*rac*-2)

(*R*) T_r = 17.2 minutes, (*S*) T_r = 18.3 minutes, Method D

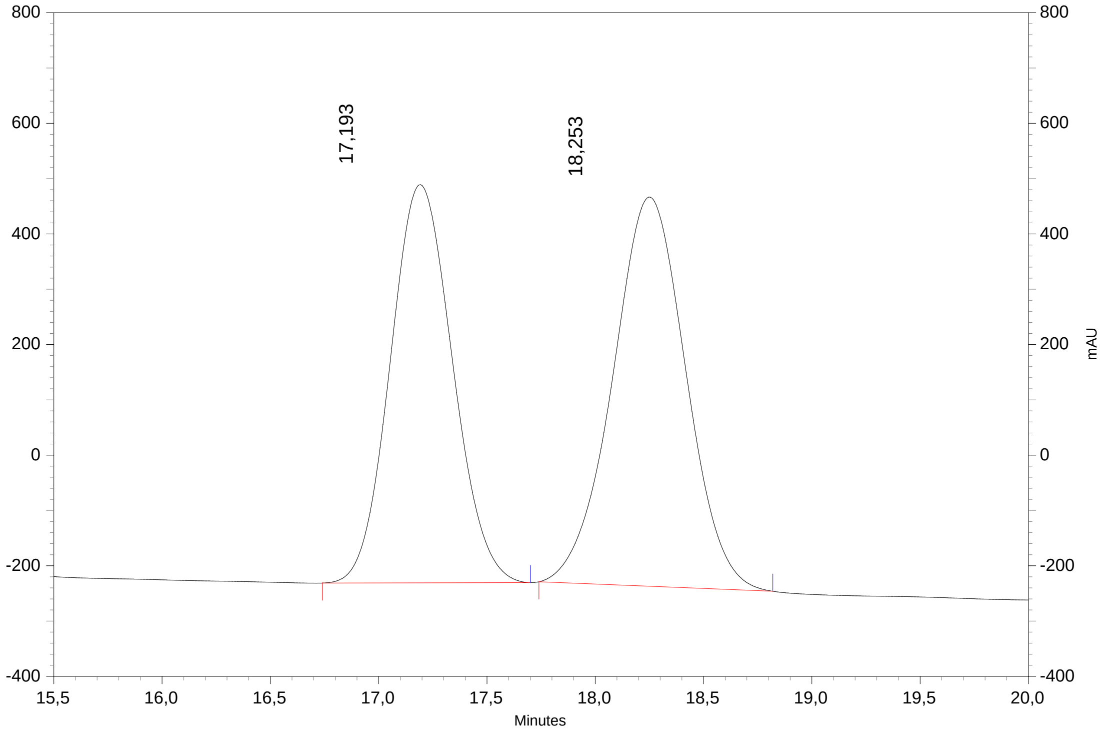

DSC

(*2S*)-*N*-((*S*)-1-phenylethyl)-2,3-dihydrobenzo-(1,4)-oxathiine-2-carboxamide (*S*,*S'*-6)

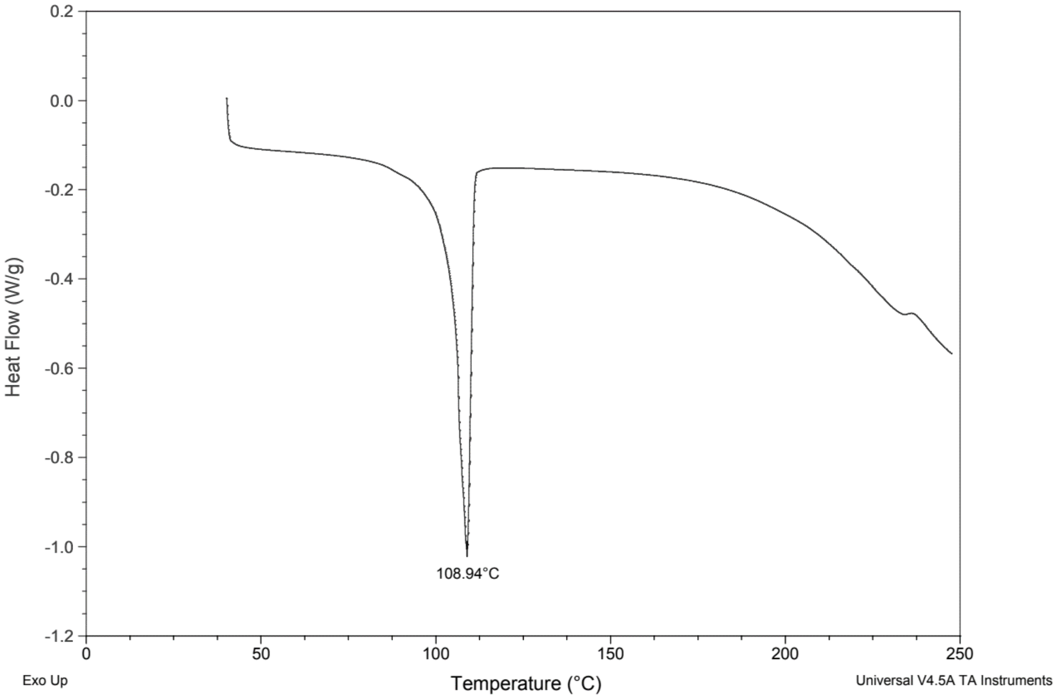

(*2S*)-*N*-((*S*)-1-phenylethyl)-2,3-dihydrobenzo-(1,4)-oxathiine-3-carboxamide (*S*,*S'*-10)

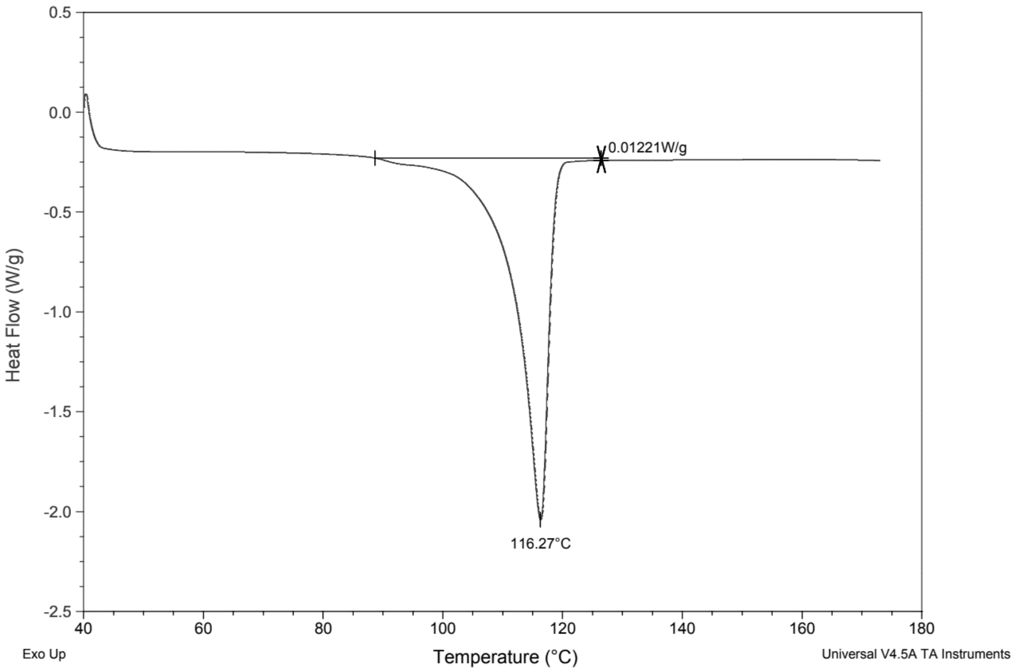

(*2R*)-N-((*S*)-1-phenylethyl)-2,3-dihydrobenzo-(1,4)-oxathiine-3-carboxamide (*R*,*S'*-10)

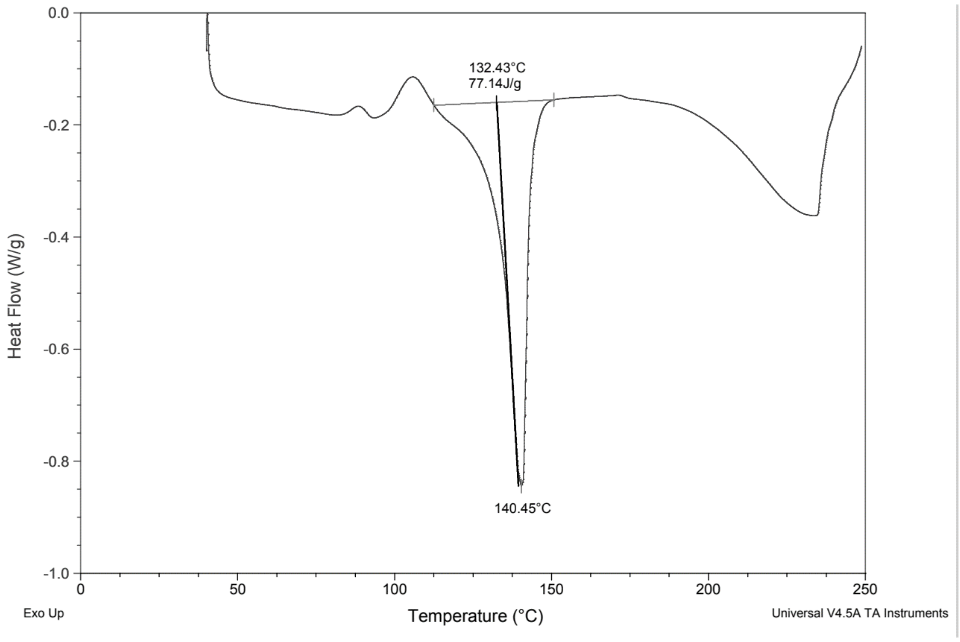

(2*S*)-2,3-dihydrobenzo-(1,4)-oxathiine-2-carboxylic acid (*S*-1) and (2*R*)-2,3-dihydrobenzo-(1,4)-oxathiine-2-carboxylic acid (*R*-1)

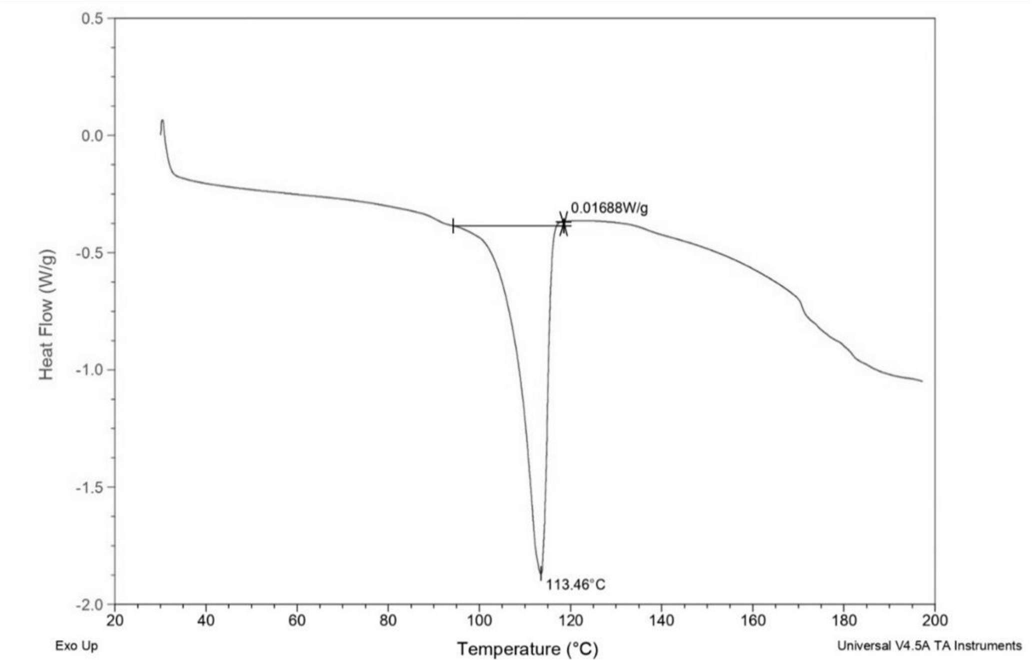

2,3-dihydrobenzo-(1,4)-oxathiine-2-carboxylic acid (*rac*-2)

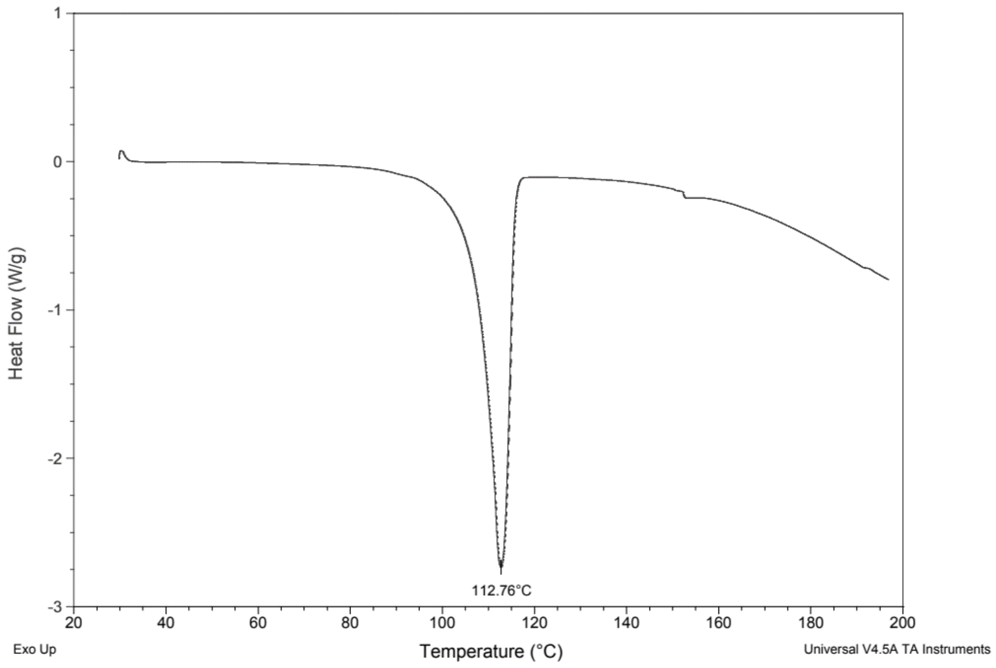

HIGH-RESOLUTION MASS ANALYSIS

(2*S*)-N-((*S*)-1-phenylethyl)-2,3-dihydrobenzo-(1,4)-oxathiine-2-carboxamide (*S,S'*-6)

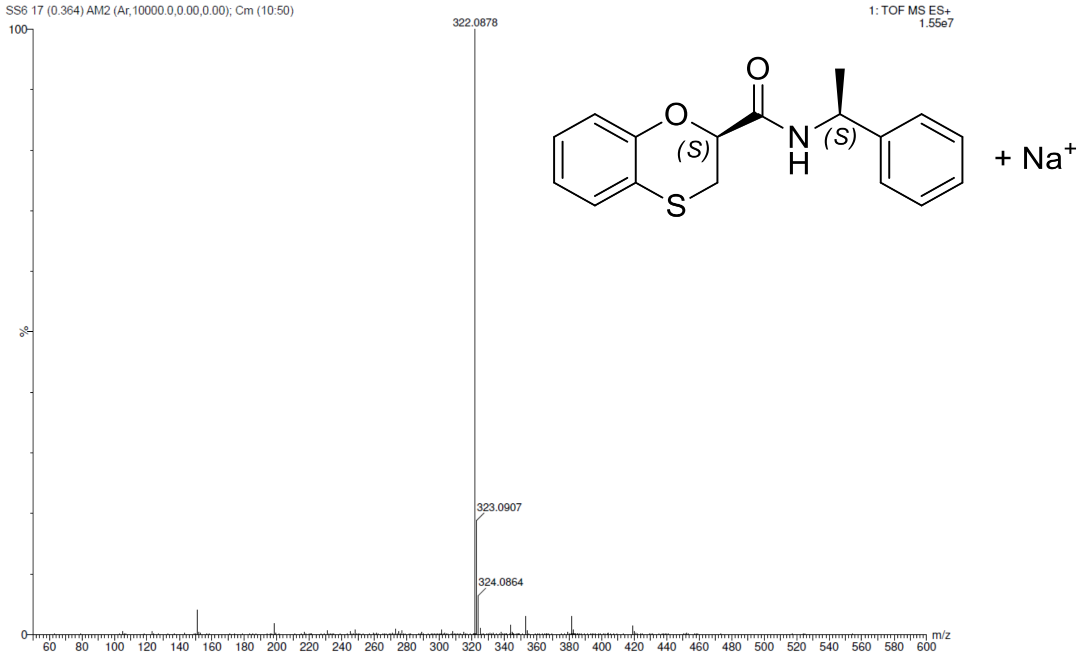

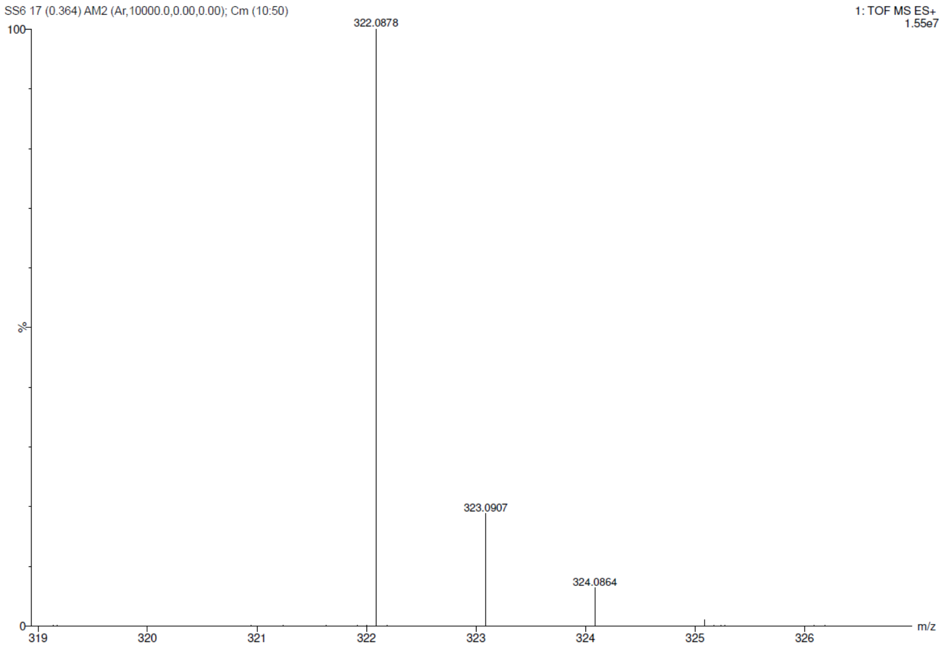

Single Mass Analysis
Tolerance = 5.0 PPM / DBE: min = -5.0, max = 300.0
Element prediction: Off
Number of isotope peaks used for i-FIT = 7

Monoisotopic Mass, Even Electron Ions
1 formula(e) evaluated with 1 results within limits (all results (up to 1000) for each mass)
Elements Used:
C: 17-17 H: 17-18 N: 1-1 O: 2-2 Na: 0-2 S: 1-1

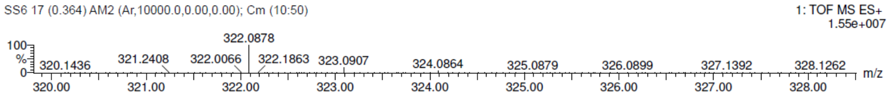

Minimum:				-5.0				
Maximum:		5.0	5.0	300.0				
Mass	Calc. Mass	mDa	PPM	DBE	i-FIT	Norm	Conf(%)	Formula
322.0878	322.0878	0.0	0.0	9.5	1482.9	n/a	n/a	C17 H17 N O2 Na S

(2*R*)-*N*-((*S*)-1-phenylethyl)-2,3-dihydrobenzo-(1,4)-oxathiine-2-carboxamide (*R*,*S'*-6)

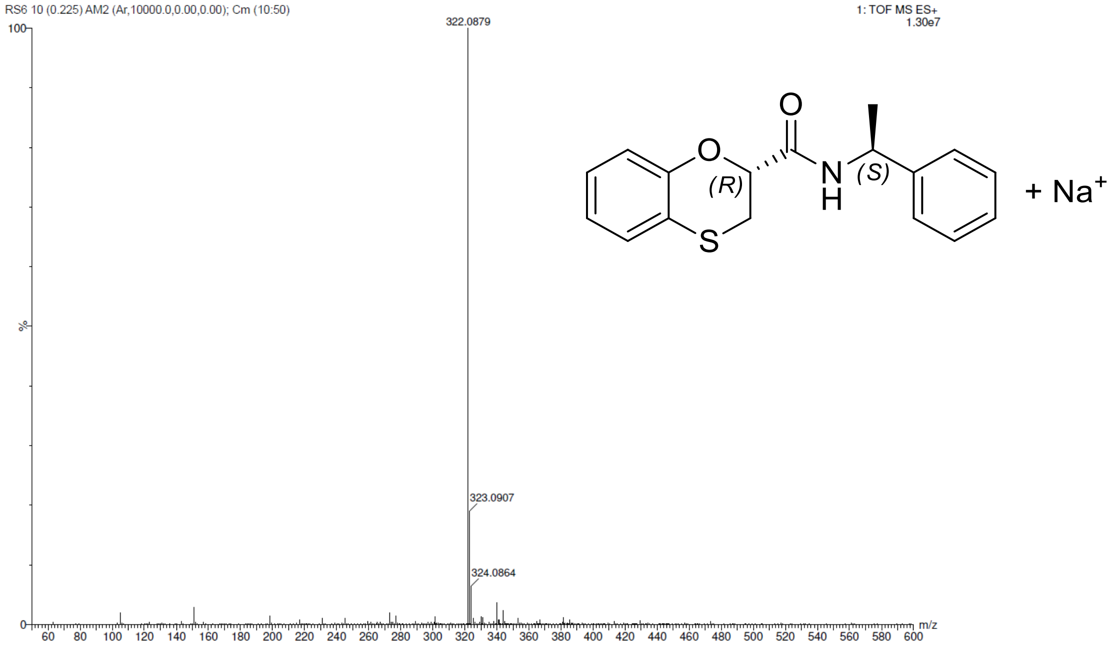

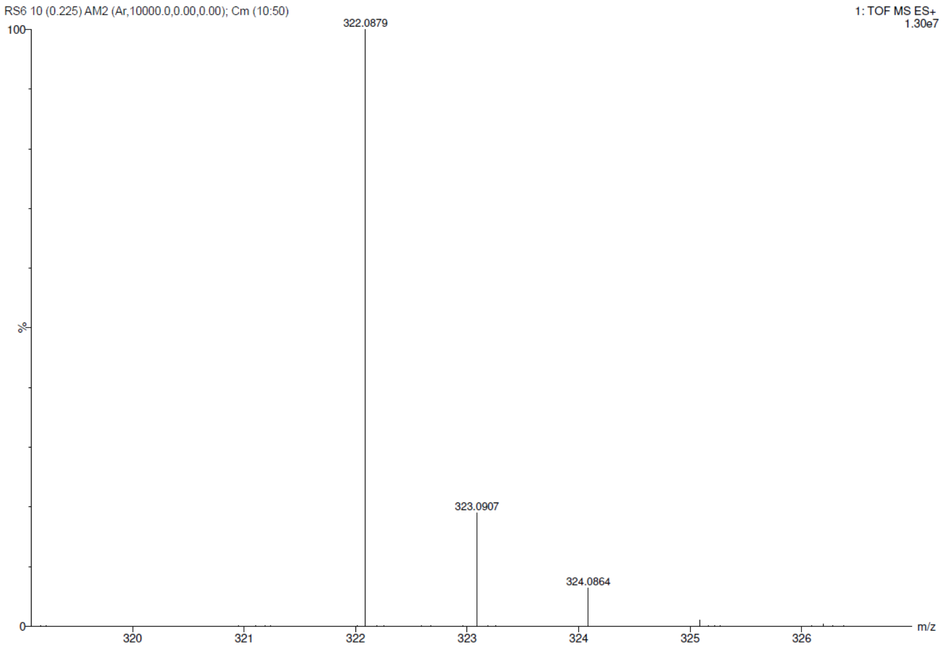

Single Mass Analysis
Tolerance = 5.0 PPM / DBE: min = -5.0, max = 300.0
Element prediction: Off
Number of isotope peaks used for i-FIT = 7

Monoisotopic Mass, Even Electron Ions
1 formula(e) evaluated with 1 results within limits (all results (up to 1000) for each mass)
Elements Used:
C: 17-17 H: 17-18 N: 1-1 O: 2-2 Na: 0-2 S: 1-1

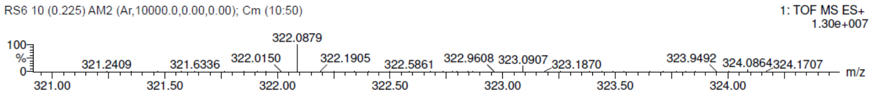

Minimum:				-5.0				
Maximum:		5.0	5.0	300.0				
Mass	Calc. Mass	mDa	PPM	DBE	i-FIT	Norm	Conf(%)	Formula
322.0879	322.0878	0.1	0.3	9.5	517.6	n/a	n/a	C17 H17 N O2 Na S

(2*S*)-2,3-dihydrobenzo-(1,4)-oxathiine-2-carboxylic acid (*S*-1)

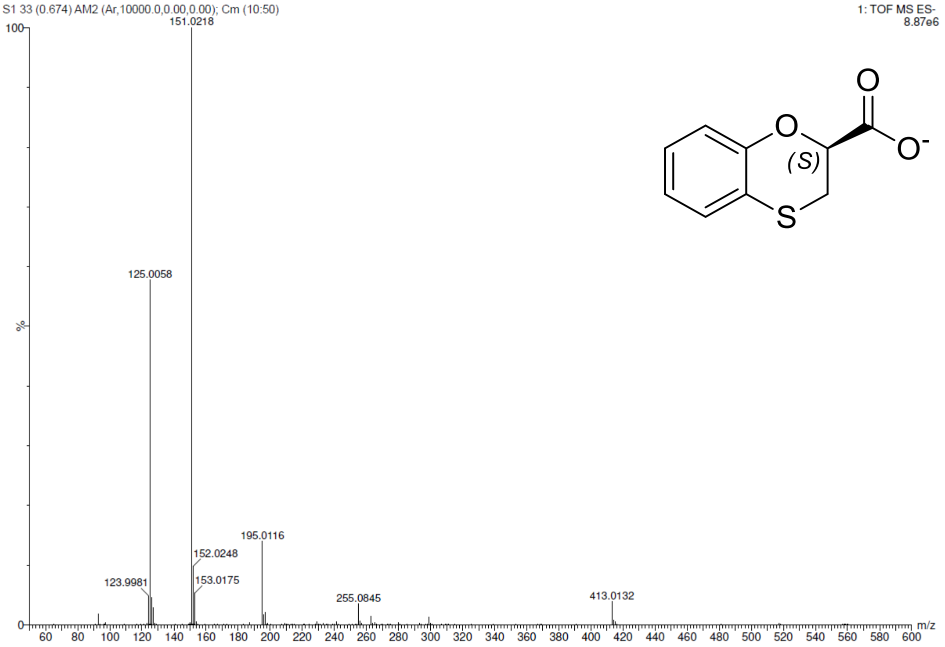

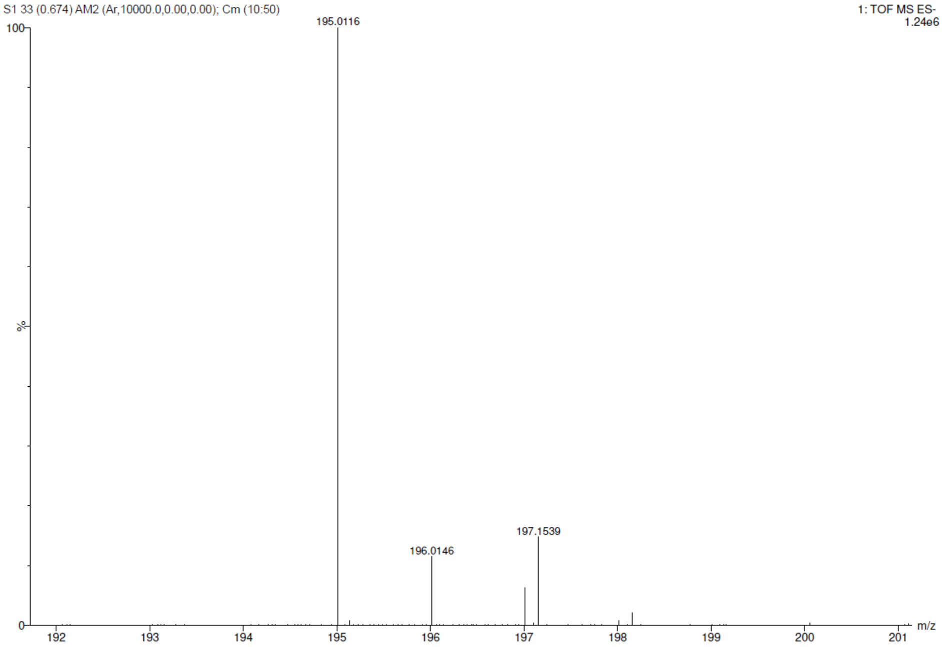

Single Mass Analysis
Tolerance = 5.0 PPM / DBE: min = -5.0, max = 300.0
Element prediction: Off
Number of isotope peaks used for i-FIT = 7

Monoisotopic Mass, Even Electron Ions
3 formula(e) evaluated with 1 results within limits (all results (up to 1000) for each mass)
Elements Used:
C: 9-9 H: 7-8 O: 3-3 Na: 0-2 S: 1-1
S1 33 (0.674) AM2 (Ar,10000.0,0.00,0.00); Cm (10:50)

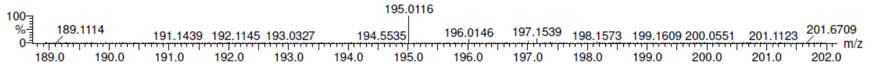

Minimum:				-5.0				
Maximum:		5.0	5.0	300.0				
Mass	Calc. Mass	mDa	PPM	DBE	i-FIT	Norm	Conf(%)	Formula
195.0116	195.0116	0.0	0.0	6.5	1892.6	n/a	n/a	C9 H7 O3 S

(2*R*)-2,3-dihydrobenzo-(1,4)-oxathiine-2-carboxylic acid (*R*-1)

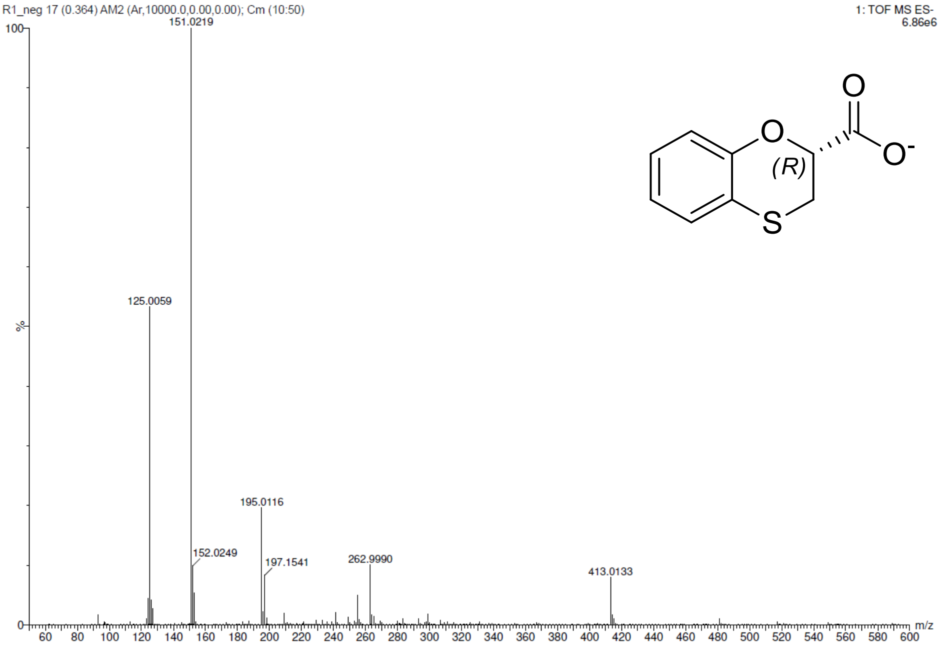

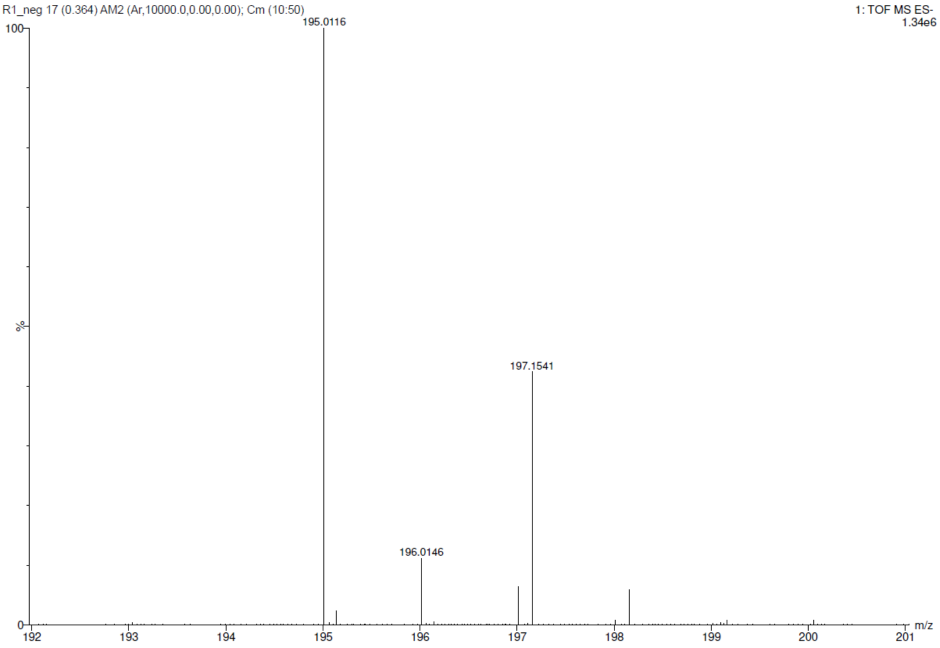

Single Mass Analysis
Tolerance = 5.0 PPM / DBE: min = -5.0, max = 300.0
Element prediction: Off
Number of isotope peaks used for i-FIT = 7

Monoisotopic Mass, Even Electron Ions
3 formula(e) evaluated with 1 results within limits (all results (up to 1000) for each mass)
Elements Used:
C: 9-9 H: 7-8 O: 3-3 Na: 0-2 S: 1-1

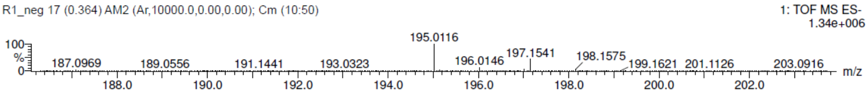

Minimum:				-5.0				
Maximum:		5.0	5.0	300.0				
Mass	Calc. Mass	mDa	PPM	DBE	i-FIT	Norm	Conf(%)	Formula
195.0116	195.0116	0.0	0.0	6.5	2052.4	n/a	n/a	C9 H7 O3 S

(2*S*)-*N*-((*S*)-1-phenylethyl)-2,3-dihydrobenzo-(1,4)-oxathiine-3-carboxamide (*S*,*S'*-10)

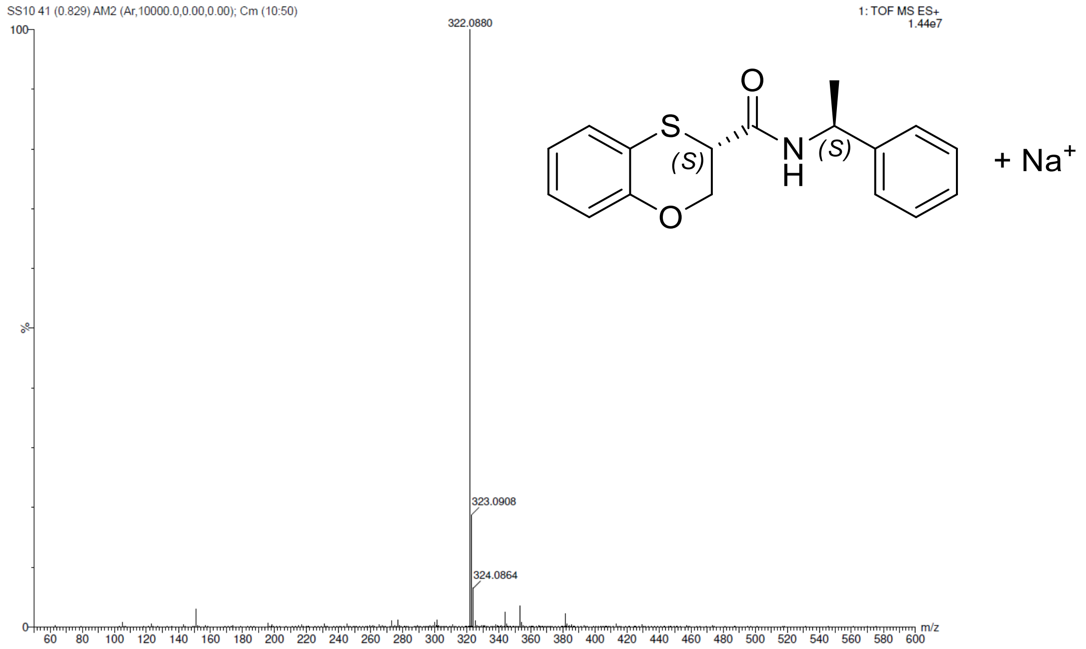

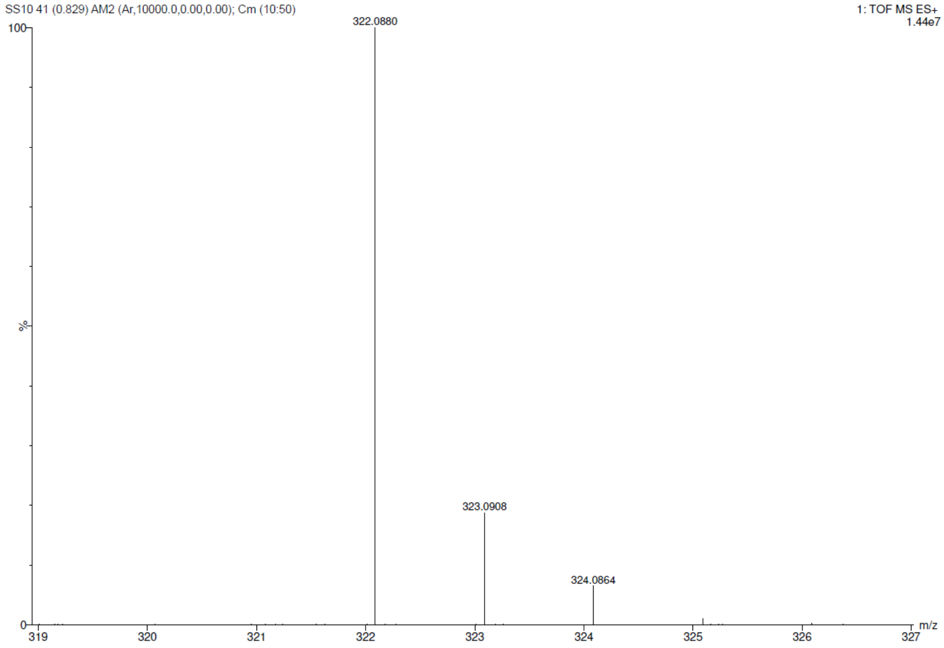

Single Mass Analysis
Tolerance = 5.0 PPM / DBE: min = -5.0, max = 300.0
Element prediction: Off
Number of isotope peaks used for i-FIT = 7

Monoisotopic Mass, Even Electron Ions
1 formula(e) evaluated with 1 results within limits (all results (up to 1000) for each mass)
Elements Used:
C: 17-17 H: 17-18 N: 1-1 O: 2-2 Na: 0-2 S: 1-1

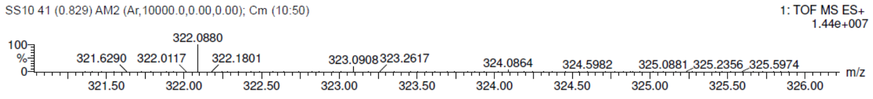

Minimum:				-5.0				
Maximum:		5.0	5.0	300.0				
Mass	Calc. Mass	mDa	PPM	DBE	i-FIT	Norm	Conf(%)	Formula
322.0880	322.0878	0.2	0.6	9.5	912.9	n/a	n/a	C17 H17 N O2 Na S

(2*R*)-*N*-((*S*)-1-phenylethyl)-2,3-dihydrobenzo-(1,4)-oxathiine-3-carboxamide (*R*,*S'*-10)

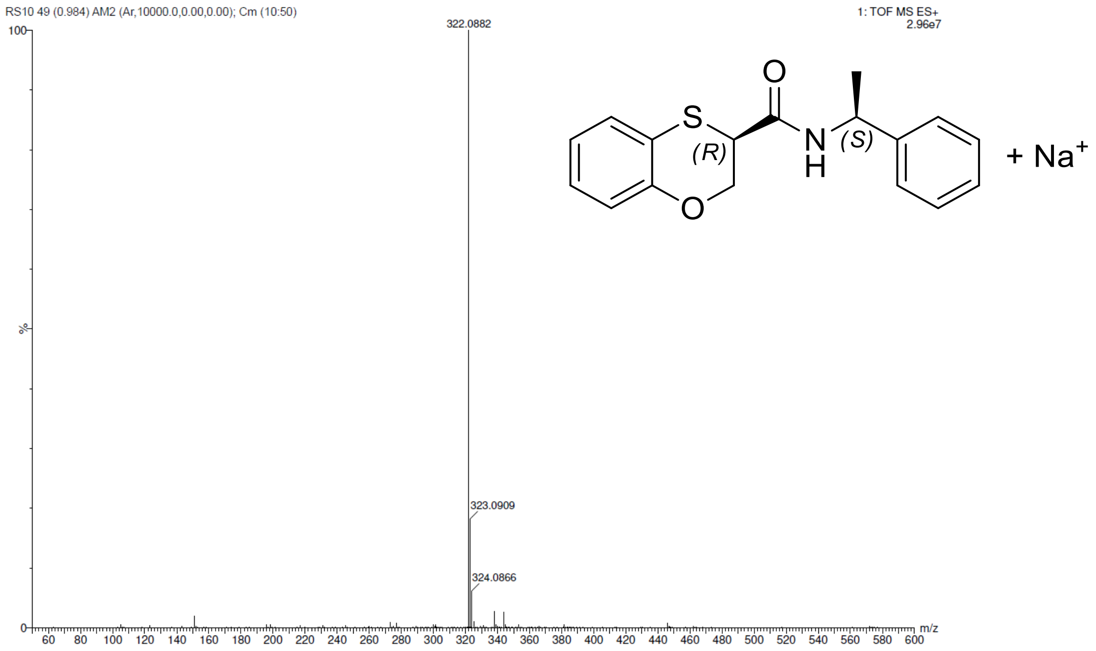

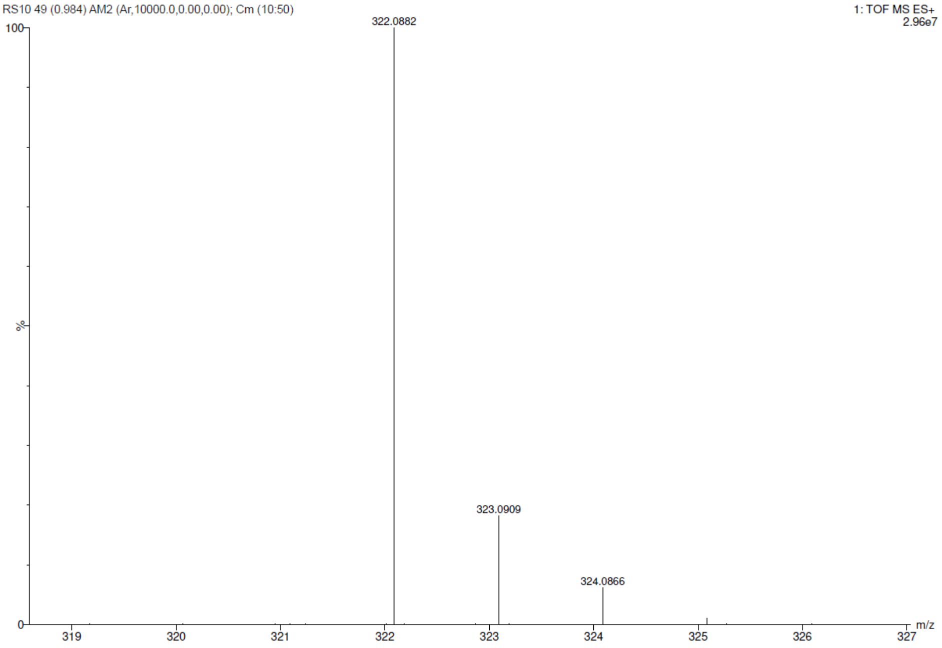

Single Mass Analysis
Tolerance = 5.0 PPM / DBE: min = -5.0, max = 300.0
Element prediction: Off
Number of isotope peaks used for i-FIT = 7

Monoisotopic Mass, Even Electron Ions
1 formula(e) evaluated with 1 results within limits (all results (up to 1000) for each mass)
Elements Used:
C: 17-17 H: 17-18 N: 1-1 O: 2-2 Na: 0-2 S: 1-1

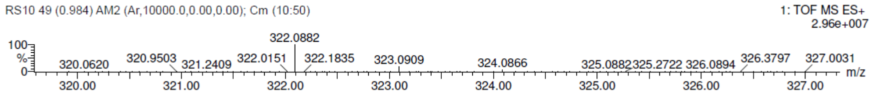

Minimum:				-5.0				
Maximum:		5.0	5.0	300.0				
Mass	Calc. Mass	mDa	PPM	DBE	i-FIT	Norm	Conf(%)	Formula
322.0882	322.0878	0.4	1.2	9.5	1193.9	n/a	n/a	C17 H17 N O2 Na S

2,3-dihydrobenzo-(1,4)-oxathiine-2-carboxylic acid (*rac*-2)

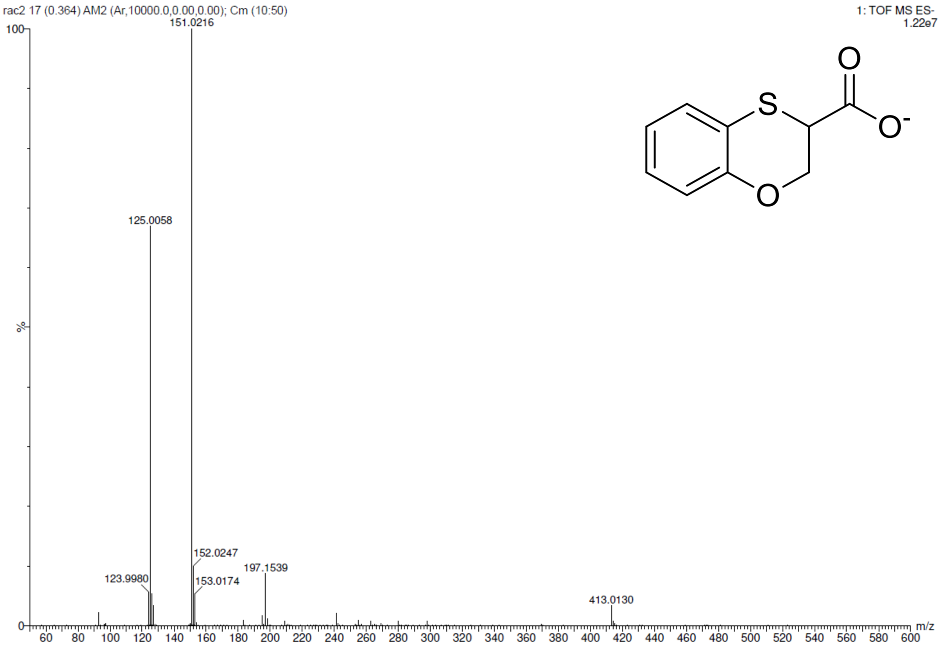

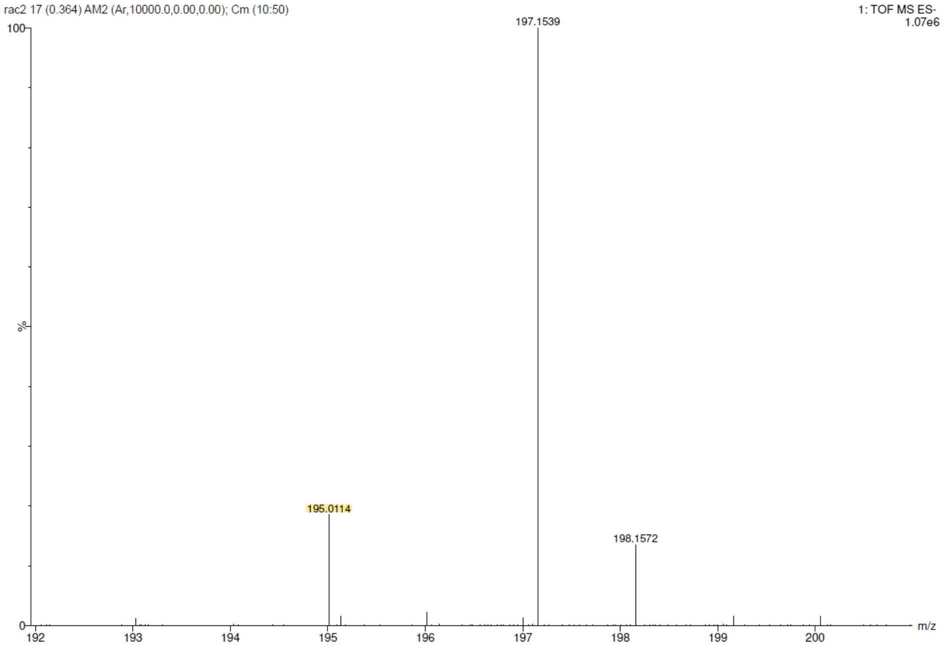

Single Mass Analysis
Tolerance = 5.0 PPM / DBE: min = -5.0, max = 300.0
Element prediction: Off
Number of isotope peaks used for i-FIT = 7

Monoisotopic Mass, Even Electron Ions
3 formula(e) evaluated with 1 results within limits (all results (up to 1000) for each mass)
Elements Used:
C: 9-9 H: 7-8 O: 3-3 Na: 0-2 S: 1-1

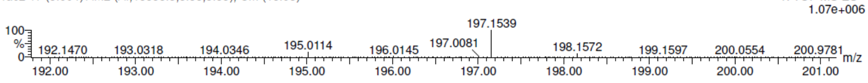

Minimum:				-5.0				
Maximum:		5.0	5.0	300.0				
Mass	Calc. Mass	mDa	PPM	DBE	i-FIT	Norm	Conf(%)	Formula
195.0114	195.0116	-0.2	-1.0	6.5	1856.1	n/a	n/a	C9 H7 O3 S
